# The problem of frailty caused by acute infection and future health management strategies to improve frailty

**DOI:** 10.3389/fpubh.2025.1735577

**Published:** 2026-01-20

**Authors:** Guihua Li, Yue Zhao, Wenhui Gu, Qianqian Wang, Xinyi Lu, Xinlei Miao

**Affiliations:** 1 Health Management Center, The Second Hospital of Dalian Medical University, Dalian, Liaoning, China; 2 Department of Cardiovascular Ultrasound, The Second Hospital of Dalian Medical University, Dalian, Liaoning, China; 3 Peking University Third Hospital, Beijing, China; 4 Research Center, The Second Hospital of Dalian Medical University, Dalian, Liaoning, China; 5 Dalian Institute of Chemical Physics, Chinese Academy of Sciences, Dalian, Liaoning, China

**Keywords:** COVID-19 pandemic, frailty, health improvement, health management, interrupted time series

## Abstract

**Background:**

We aimed to analyze changes in frailty associated with long-COVID, while providing effective health management measures to improve frailty.

**Methods:**

We conducted a 4-month follow-up cohort study involving 2,471 participants to analyze changes in body frailty after the prevalence of COVID-19 in China. We performed interrupted time series analysis to estimate the impact of acute infection on the changes in frailty. The time-dependent COX model was considered to assess the association between frailty status and infection, and sensitivity analysis was performed to verify the stability of the results. In addition, we established a traditional Cox model to analyze the relationship between healthy behaviors and infections, aiming to improve health management and reduce frailty.

**Results:**

There were significantly elevated trend changes in the frailty index compared to the prepandemic period in the total population (+0.029[0.016, 0.041], *p* < 0.001), and the frailty index was found to be higher in female individuals and people aged over 65 years. Participants with moderate frailty (HR = 1.19, 95% CI: 1.04–1.35, *p* < 0.001) and severe frailty (HR = 1.34, 95%CI: 1.15–1.56, *p* < 0.001) had a significantly higher hazard of infection than those with mild frailty. Long-term health monitoring indicated that positive mood, appropriate physical activities, sufficient intake of grains and vegetables, and reduced intake of sugary drinks can improve frailty and ultimately reduce the risk of infection.

**Conclusion:**

In this study, it was found that the population generally became more frail after the pandemic, and frailty increases the risk of acute reinfection. Therefore, it is necessary to carry out health management strategies to improve frailty.

## Introduction

Coronavirus disease 2019, commonly referred to as COVID-19, first emerged in 2019 and has persisted for nearly 3 years, resulting in a global pandemic. On 5 May 2023, the World Health Organization declared an end to the public health emergency of international concern (1). However, this does not imply that acute conditions are no longer a threat to population health. Some studies have confirmed that the majority of infected people develop mild to moderate illness and recover without requiring hospitalization. Typically, recovery occurs gradually after 1 week or may even take up to 14 days (2–4). After the acute infection, a proportion of people experience physical symptoms, such as cough, fatigue, or poor sleep quality. Unfortunately, these symptoms can persist (5, 6) and are often known as post-acute syndrome (PCS) or long-COVID (5).

Recently, frailty has gained attention in the field of health management. Frailty is an age-related clinical condition characterized by a decline in physical and physiological functions (6). The literature suggests that frailty is closely related to chronic inflammation and insulin resistance, thereby accelerating the occurrence of cardiovascular and other diseases (7). Furthermore, another mechanism identified is the association between hypoglycemia and frailty, which can increase the risk of cardiovascular diseases (8). In summary, previous studies have found that acute infection may have an impact on physical functions such as pulmonary, neuropsychiatric, and cardiovascular health, particularly among older people. However, there is still limited evidence on the relationship between frailty and long-COVID due to the complexities surrounding frailty. Therefore, detailed information about frailty in the general population after acute infection is necessary to inform future health management strategies.

We aimed to analyze changes in frailty before and after the acute infection and to identify health management measures for addressing frailty in the future.

## Materials and methods

### Study design

In this study, pandemic refers to COVID-19, which is defined as an acute infection, that is, the diagnosis of COVID-19 infection. Based on the COVID-19 study, an add-on study to the Dalina Health Management Cohort (DHMC) (ChiCTR2300073363) observed the health status after infection of the health examination population in the northeast of China. DHMC is an open cohort study established by personal physical examination of the Second Hospital of Dalian Medical University in China, which is a longitudinal survey launched in 2014. DHMC was designed to collect the results of personal physical examination included laboratory test results (biochemical examination), auxiliary examinations (ultrasonic testing, etc.), health questionnaire (demographic variables, lifestyles, disease history, medication history, nutritional status, psychological situation), and evaluate population health status, and then conduct health management.

We included all individuals (aged ≥18 years) who underwent two health assessments. The first assessment was conducted at some point during the year 2022. The second assessment took place between 1 January and 30 April 2023. Therefore, the approximate interval between the first and second assessments for any given participant ranged from approximately 9 to 16 months, with a typical interval of approximately 1 year. We only included individuals who completed both assessments. We excluded 1,321 individuals with incomplete information and ultimately included 2,471 participants (43.10% female) in a self-control study on the changes in physical health before and after COVID-19 infection. Analyses were performed using the entire data set (N = 2,471) and one subset (N1 = 2,264, who were infected after 1 December 2022, and had undergone physical examination). The flowchart is shown in Supplementary Figure S1. This study was conducted in accordance with the principles of the Declaration of Helsinki, and the health management cohort study and its add-on study were approved by the Medical Ethical Committee of the Second Hospital of Dalian Medical University. This study has informed consent obtained from all participants.

·

### Frailty and outcome measures

This study updated the components of frailty by combining Fried, Rockwood, and Mitnitski (9–11). We used a combination of self-reported health and routine examination results to measure the frailty index (FI) in order to estimate frailty. FI is a comprehensive indicator that integrates information from biomarkers, medical history, living habits, etc. In our study, FI was measured for each participant separately using 54 items, 14 categories (Supplementary Table S1) in each medical examination year. Each item in each category is defined as a binary classification variable according to the diagnostic criteria (12). The score of the sum of the items in each category≥1 is defined as a new binary classification variable indicating having a health deficit; otherwise, there is no health defect. For each participant, FI is calculated as the proportion of health attributes that a person has in a deficit state, ranging from 0 (no health deficit) to 1 (health deficit). Finally, we divided the degree of frailty according to the tri-sectional quantiles of FI, namely mild frailty, moderate frailty, and severe frailty. We defined acute infection as the outcome based on the questionnaire “whether you have confirmed COVID-19 infection through antigen self-test or nucleic acid test recently” reported by the participants during physical examination.

### Covariates definition

All variables were defined as binary classification variables. Smoking and alcohol consumption were categorized as yes (having related experience) or no (not having related experience). Metabolic equivalent (MET) was calculated from the relevant physical activity types and time reported by participants, and MET ≤ 3 was defined as low physical activity, MET > 3 and ≤ 6 was defined as moderate physical activity, MET > 6 was defined as vigorous physical activity. (13). We defined individuals under the age of 60 who sleep for more than 7 h and those over 60 years who sleep for between 5 and 8 h as normal sleep (14). Having good tooth brushing habits means brushing teeth twice or more a day. The dietary nutrition standards are evaluated according to the provisions of the Chinese Dietary Guidelines 2022 (15), which include a comprehensive evaluation of 24 h of grains, meat, eggs, aquatic products, tubers, vegetables, fruits, nuts, soy products, white water, and milk. The evaluation criteria are the same as the rules for calculating FI, and ultimately, the population that meets dietary standards is considered to have a dietary score of 0. The drinking amount of tea is defined as 2–3cups per day as mild intake, 4–5 cups as moderate intake, 6–8 cups as excessive intake, and more than 8 cups as severe intake, based on the reference of less than one cup (250 mL) per day. The soda water is defined as no intake per week, 1–5 cups per week, 6–9 cups per week, and more than 10 cups per week as reference, mild intake, moderate intake, and excessive intake, respectively. Compared to not drinking coffee, we defined 1–2 cups per week as mild intake, 3–5cups per week as moderate intake, and more than 5 cups as excessive intake. Mild intake of sugar-sweetened beverages was defined as 1–2 bottles per week, 3–4 bottles of sugar-sweetened beverages as moderate intake, and more than 4 bottles per week as excessive intake. There were 11 items about psychological stress for the subjects, and those with a total score of 0 were defined as optimistic about psychological stress. We defined nodules of BI-RADS 3 and above in thyroid ultrasound and C-TI-RADS 3 and above in breast ultrasound as having nodules. Ultrasound diagnosis of any degree of fatty liver was defined as having fatty liver. Pulmonary nodules and lung inflammation were defined through the diagnosis of low-dose computed tomography (LDCT). Cervical vascular abnormalities are defined as the phenomenon of plaques, narrowing, and thickening of the intima media in cervical blood vessels. The body mass index (BMI) was calculated by the ratio of weight (kg) and the square of height (m^2^), and a BMI between 18.5 kg/m2 and 24 kg/m2 is considered normal. The definition of having a family history, personal history, and medication history was that the participant had any related historical event.

### Statistics

We preliminarily described the characteristics of the data, which included continuous normal variables summarized by means and SDs, and medians and IQRs for continuous non-normal variables. Differences in baseline characteristics between groups were compared using independent samples t-tests or Mann–Whitney U-tests for continuous variables (as appropriate based on distribution), and chi-square tests or Fisher’s exact tests for categorical variables.

We used the Kaplan–Meier method to estimate the uninfected rate of COVID-19 and used the log-rank test to compare the rates by the degree of frailty. The Cox proportional hazards model (Cox PH) was tested using the cox.zph function in the R Survival package. We adjusted three models of the Cox PH to assess the association between frailty status and infection. Analyses were conducted stratified by frailty category. Within each stratum, regression was used to assess the association of health behaviors with the likelihood of frailty, using the favorable level of each behavior as the reference. Frailty status was considered to have a divergent association with outcomes if they were significantly associated with COVID-19 infection, but the log2 hazard ratios had opposite signs. For the variables in the model that failed the assumption for a Cox PH model (*p* < 0.05), a time-dependent COX model was considered (as detailed in Supplementary Figure S2). We use the tt parameter and the log conversion to construct the interaction term of the variables that time transform in order to solve the problem of coefficients changing over time. The model is specified as:

tt=function(x,t,…)x∗log(t+20)

where x is the covariate with time-varying effects, and t is the analysis time.

Subsequently, we still established three traditional Cox regression models to analyze the association between frailty and health behaviors. The first model was a crude model with unadjusted. The minimal adjusted model was adjusted sex and age. The last model was adjusted for vaccination based on the minimal model.

We performed an interrupted time series analysis (ITSA) to estimate the impact of COVID-19 infection on the change in frailty. ITSA is a common method in public health, which tests the effectiveness of an intervention by controlling the upward or downward trend of outcome variables before intervention, including changes in levels and trends before and after interruption. First, we used least squares estimation for the Breusch–Godfrey test of independent variables and hysteresis residuals. Second, we used segmented regression to establish a model to fit the impact of a sudden COVID-19 incident on frailty. We compare outcomes before and after a policy intervention using two time points. The model is specified as:

$$Y_{t}=\beta_{0}+\beta_{1}\text{time}+\beta_{2}\mathrm{int}\text{ervention}+\beta_{3}\text{post}$$

$Y_{t}$ is the value of FI in time t; time is a continuous variable indicating the time span, where the first observation (2022.1.1) was defined 0, and the last observation (2023.4.31) was defined as 2,471; intervention is a binary variable indicating whether the participant was infected; post is a continuous number beginning with 0 after the infection on 1 January 2023. $\;\beta_{0}$ estimates the FI at the beginning of the time series. $\beta_{1}$ estimates the preinfection trend of FI. $\beta_{2}$ estimates the immediate change in the level of FI after infection. $\beta_{3}$ estimates the trend of FI after infection.

A sensitivity analysis was conducted using a segment time-dependent Cox model. We use a hierarchical function for time and divide it into two periods based on the proportional risk assumption graph: 0–14 days and after 14 days. Then we fit and explore the impact of frailty on the occurrence of COVID-19 infection based on the inflection point.

All statistics and data analysis were performed in Stata SE15 and R 4.3.0. Estimated effects with 95% CIs were calculated, and a *p*-value of <0.05 was considered statistically significant.

## Results

### Baseline information

The baseline characteristics are presented in Table 1. A total of 793 female participants and 743 male participants were acute positive. Compared to an uninfected female, an infected female tends to be younger, more alcohol consumption, have a worse psychological status, have a family medical history, have higher FI, and be of moderate or severe frailty status (*p* < 0.05). Compared to an uninfected male, an infected male tends to be younger, non-smoking, non-alcohol consumption, poor sleeping, and have a poor psychological status, having a personal and family medical history, higher FI, and being of moderate or severe frailty status (*p* < 0.05).

**Table 1:** Baseline characteristics of the participants (*N* = 2,471).

Variables	Female (*n* = 1,065)			Male (*n* = 1,406)		
	Infected (*n* = 793)	Uninfected (*n* = 272)	*P*-value	Infected (*n* = 743)	Uninfected (*n* = 663)	*P*-value
Age (year)			**<0.001**			**<0.001**
18–44	539(68.00%)	144(52.90%)		362(48.70%)	247(37.30%)	
45–64	234(29.50%)	93(34.20%)		358(48.20%)	377(56.90%)	
65 and above	20(2.50%)	35(12.90%)		23(3.10%)	39(5.90%)	
Smoking	7(0.90%)	3(1.10%)	0.745	355(47.80%)	356(53.70%)	**0.027**
Alcohol consumption	141(17.80%)	32(11.80%)	**0.02**	515(69.30%)	384(57.90%)	**<0.001**
Sleeping	164(20.90%)	41(15.80%)	0.069	127(17.30%)	78(12.10%)	**0.007**
Physical activity	44(5.60%)	18(6.70%)	0.508	36(4.90%)	19(2.90%)	0.057
Diet	413(52.10%)	141(51.80%)	0.945	401(54.00%)	384(57.90%)	0.137
Tooth brushing	96(12.10%)	44(16.20%)	0.086	291(39.20%)	261(39.40%)	0.939
Psychological status	568(71.60%)	165(60.70%)	**0.001**	468(63.00%)	341(51.40%)	**<0.001**
Frailty index	0.30 ± 0.09	0.29 ± 0.09	**0.006**	0.37 ± 0.10	0.36 ± 0.09	**0.002**
Frailty status			**0.005**			**0.004**
Mild frailty	373(47.04%)	159(58.46%)		143(19.25%)	174(26.25%)	
Moderate frailty	311(39.22%)	83(30.51%)		298(40.11%)	258(38.91%)	
Severe frailty	109(13.74)	30(11.03%)		302(40.64%)	231(34.84%)	

Bold font indicates statistically significant differences (*P* < 0.05).

### The changes in FI before and after acute infection

**Figure 1:**
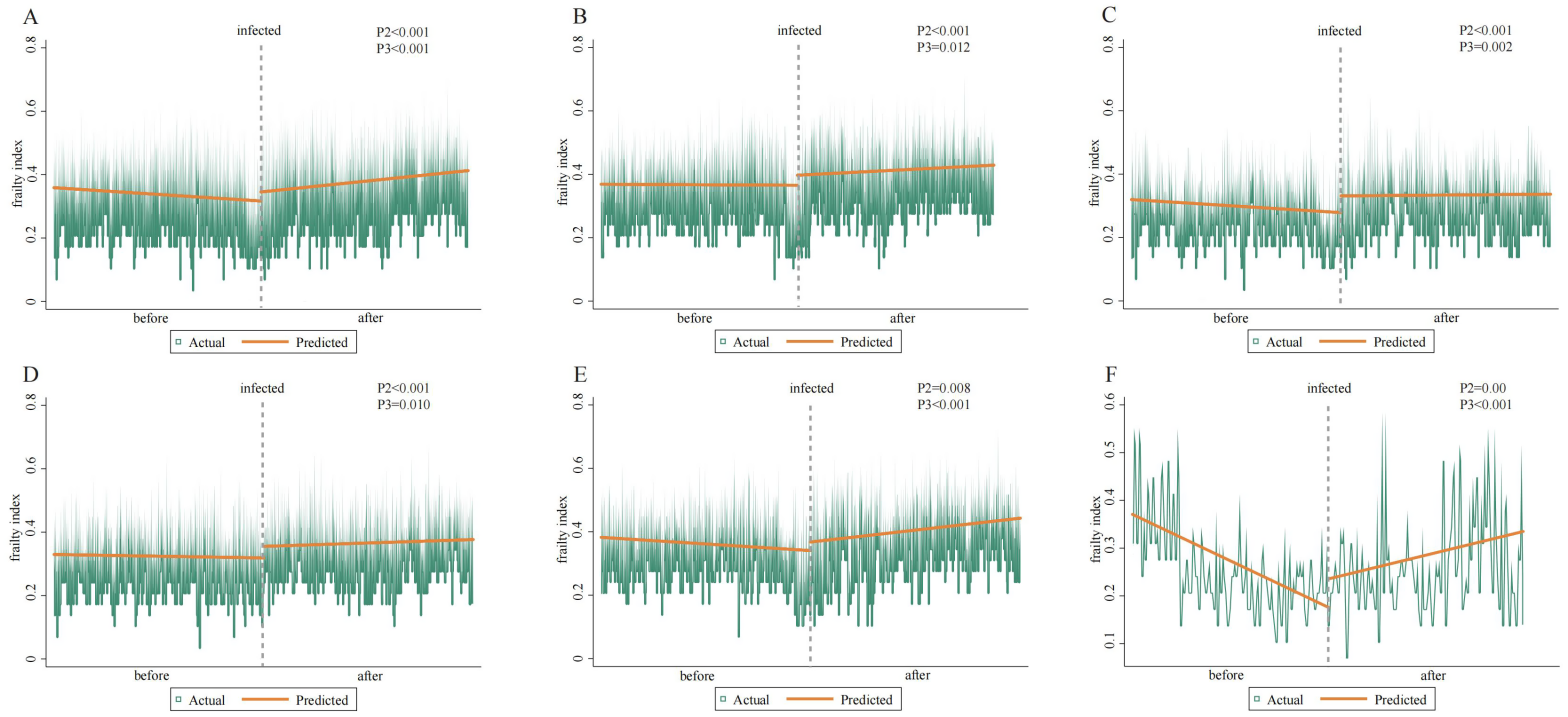
Changes in the frailty index before and after infection. Panels **(A–C)** depict the changes in the total population, males, and females, respectively, while panels **(D–F)** show the changes in the under-45, 45–64, and over-65 age groups.

A significant increasing trend in the frailty index (FI) was observed in the overall population following acute infection, with a mean change of +0.029 (95% CI: 0.016, 0.041; *p* < 0.001). At baseline, males exhibited a higher FI (0.369) than females (0.320). However, following infection, the immediate increase in FI was more pronounced in females (+0.053; 95% CI: 0.035, 0.070; *p* < 0.001) than in males (+0.032; 95% CI: 0.015, 0.049; *p* < 0.001). The most marked instantaneous change in FI was observed among individuals aged 45 to 65 years. The details about the change in the frailty index of the population before and after the pandemic are shown in Figure 1. Subgroup analysis based on infection status revealed that individuals who experienced an acute infection had a slower immediate change ( $\beta_{2}$ ) in FI (0.028; 95% CI: 0.013, 0.043; *p* < 0.05) compared to those who were not infected. This difference was particularly evident among adults aged over 65 years. A detailed comparison of FI changes between infected and uninfected individuals is provided in Supplementary Table S2. Furthermore, analysis of the compositional changes in the frailty index indicated that 20 biomarkers exhibited significant immediate alterations after infection in the overall population. Additional details are available in the Supplementary Figures S3–S8; Supplementary Tables S3–S6.

### The association between FI status and acute infection

Table 2 shows that moderate frailty participants and severe frailty participants had a significantly higher hazard of infection than the mild frailty participants in the adjusted model; the HR was 1.19 (95% CI: 1.04–1.35) and 1.34(95%CI: 1.15–1.56). The results did not change after using the segment time-dependent Cox model for sensitivity analysis.

**Table 2:** Association between FI status and acute infection.

Frailty	Time dependent cox			Sensitive analysis		
	Crude model	Minimal adjusted model	Adjusted model	Crude model	Minimal adjusted model	Adjusted model
Mild frailty	Ref	ref	ref	ref	ref	ref
Moderate frailty	1.05 (0.93, 1.20)	1.20 (1.06, 1.36)*	1.19 (1.04, 1.35)*	1.05 (0.93, 1.20)	1.21 (1.06, 1.37)*	1.20 (1.05, 1.36)*
Severe frailty	0.97 (0.85, 1.12)	1.34 (1.15, 1.55)*	1.34 (1.15, 1.56)*	0.97 (0.85,1.12)	1.35 (1.16, 1.57)*	1.35 (1.16, 1.57)*

* means *p*-value <0.05. A crude model had not been adjusted. A minimal adjusted model for age and sex. Adjusted model for age, sex, and number of vaccinations. The association between sex and infection in the second time period (after 14 days): HR = 0.45(95%CI: 0.34–0.58, *p* < 0.001).

### The association between healthy behavior and frailty status

**Figure 2:**
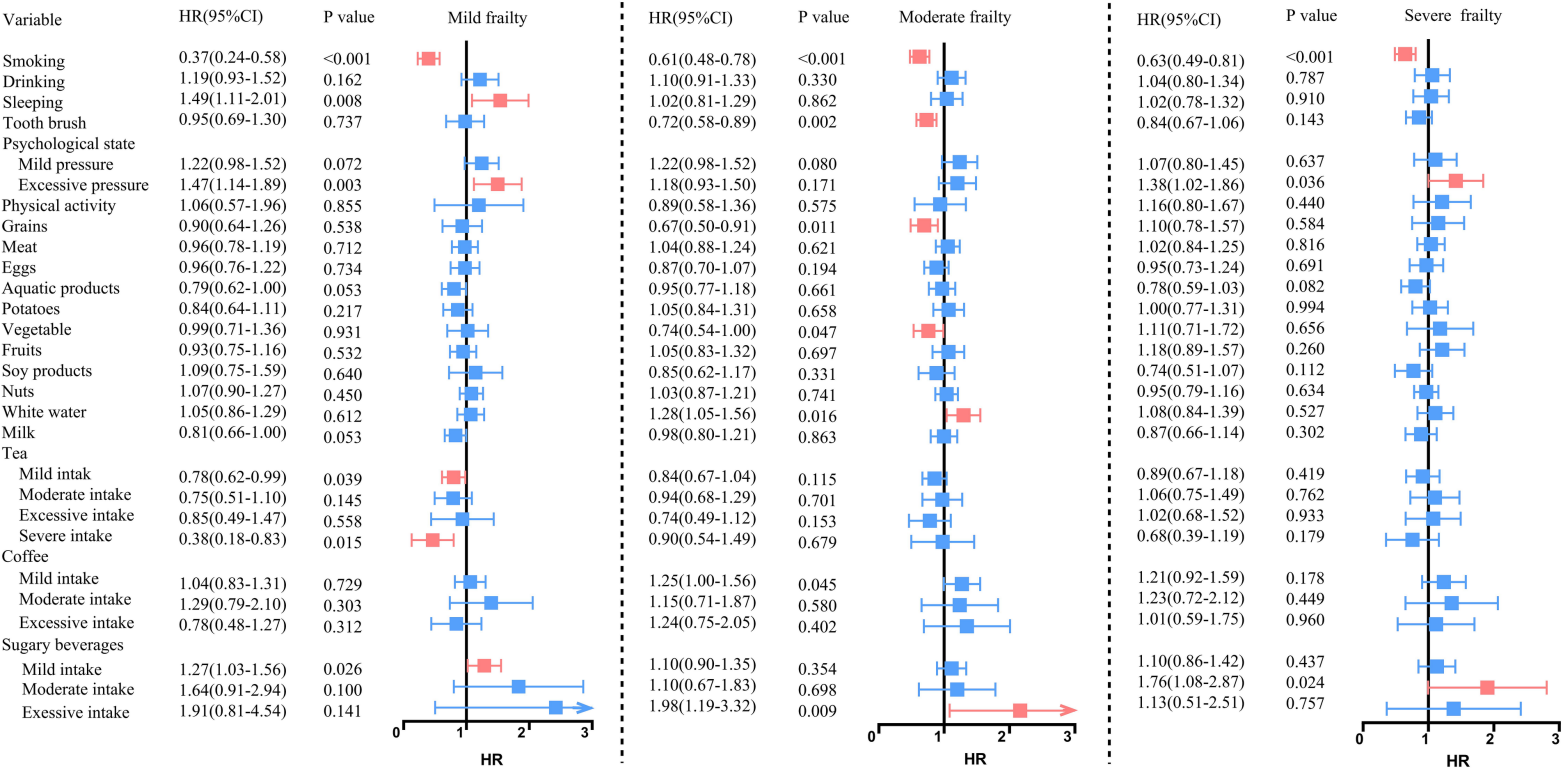
Association between healthy behavior and frailty status.

The improvement of different health behaviors varied in different frailty statuses after adjusting for age, gender, and vaccination (Figure 2). In mild frailty, the HR of 1.49 (95% CI = 1.11, 2.01) and 1.47 (1.14, 1.89) for participants with poor sleep quality and higher psychological scores. Among moderate frailty, grains and vegetables can reduce the risk of disease by approximately 70% when they meet the standard. The higher psychological score increases the risk of infection by 38% (HR = 1.38, 95% CI = 1.02, 1.86). People in severe frailty engaging in excessive physical activities actually increase infection risk by 38% (HR = 1.38, 95%CI: 1.02, 1.86). It is worth noting that regardless of the degree of frailty, smoking can reduce the risk of infection by more than 30%. Meanwhile, sugary drinks increase the risk of infection by 20–90%.

## Discussion

We conducted a 4-month follow-up study on 2,471 participants to analyze the changes in frailty after the prevalence of acute infection in December 2022 in China, confirming that acute infection will have a long-term impact on the population. The study found that people became frail after acute infection, while female individuals and people over the age of 65 years continued to be weak for a longer time. When the population is generally infected, there are instantaneous changes in various serum indicators in the body, and these positive results have not recovered for at least 4 months, which poses a risk of recurrent infection, so it is necessary for the population to promptly improve their frailty. The future health management of correct healthy behaviors, such as maintaining a positive mood, engaging in regular physical activity, ensuring adequate intake of whole grains and vegetables, and reducing the consumption of sugar-sweetened beverages, could reduce the risk of infection.

Since the epidemic of acute infection, people have faced its health effects to varying degrees, and one of those is called frailty, which generally occurs before or coexists with disease (16, 17). Even in dynamic and updating research, the core elements of frailty are still recognized to include both physiological and psychological. The relationship between acute infection and frailty status is poorly understood, while our results provide evidence that the risk of acute infection increases with the degree of frailty, after adjusting for confounding factors, and the risk of infection caused by severe frailty is 1.35 times higher than mild frailty.

Frailty no longer only exists in older people but also in various age groups. Despite the unknown global prevalence of frailty, it is higher in women than men and rises with age, as observed in many studies (18, 19), and a similar sex-specific trend has been confirmed in pre-frail older people (20). This is similar to our research results, where we found that the frailty index of females is significantly higher than that of males, and this phenomenon still exists in long-term care for at least 4 months after infection, especially in 65 + elders. It was found that although the lifespan of the population has been extended, the overall years of disability have significantly increased in the study of healthy life expectancy (21). Females have lower mortality rates than males, while their unhealthy years are also higher, which may explain that females may have more frailty than males and even be more susceptible to infection after 14 days of acute infection. The studies also indicated that differences in physiological structure, hormone secretion, immune cells, social behaviors, and roles can all lead to females being frailer than males of the same age (18, 22, 23). An acute infection can induce local inflammation, and studies have shown that changes in immune cells can explain the increased neutrophil ratio and decreased lymphopenia in patients (19, 24). Our results also confirmed that the population undergoes changes in lymphocytes and neutrophils when facing acute infection, and this change still exists in the long-term recovery, especially in women, which can result in a slower recovery of the female immune system and lead to further frailty. This all suggests the need for long-term health management, especially for females, to respond to frailty changes.

Recent research results also indicate that healthy behaviors are closely related to improving the inflammatory environment in the body, including personal habits and diets (25). The results of this study found that poor sleep quality and high psychological scores, excessive physical activities, and any level of sugary drinks intake increase the risk of infection, and the standard level of grains and vegetables intake can reduce the risk of disease by approximately 70%. In this study, a higher intake of sugar-sweetened beverages was associated with an increased risk of frailty, suggesting that reducing their consumption may be advisable. Adequate consumption of protein and a good dietary pattern are considered to control inflammation and be beneficial for health (26, 27). Physical activities impact all immune cells and reduce systemic inflammation (28), and our study suggested that people undergo moderate physical activities, which is similar to some studies. In addition to improvements in lifestyle and dietary patterns, the immune system and inflammation are currently accepted as contributors to brain-related diseases, and an optimal psychological state is also essential (29) that why we also found that people with psychological health issues were also more susceptible to infection. Therefore, timely long-term health management is likely to be needed to carry out these healthy behaviors.

It was worth noting that our findings found smoking was a protective factor against pandemic infections. In animal experiments, nicotine may have an anti-inflammatory effect to reduce tissue injury in the initial stages of infection (30, 31). A study from Italy found that the risk of acute infection among former smokers was lower than that of non-smokers (32). Numerous studies have shown the harm of smoking to physical health, such as the risk of respiratory infections and various other diseases. Of course, this result may also be related to the healthy smoker effect, differences in health-seeking behavior, or residual confounding. However, there is insufficient epidemiological evidence at present to support that nicotine in cigarettes might be beneficial to protect people from acute infection. It is conflicting whether smoking causes acute infection, but there is no doubt that smoking is harmful to health. Therefore, it is necessary to further determine how tobacco products affect susceptibility to infection, and it needs to increase the evaluation of the impact of tobacco on population health in long-term health recovery.

This study supplements the evidence on the association between frailty and acute infection, and also emphasizes that people need to carry out appropriate health management to achieve the goal of health recovery in the long-term recovery after infection. While it also has some limitations. First of all, our results may underestimate the relationship between the variable factors and acute infection because of their interactions. Second, there is still controversy regarding the definition of frailty. Therefore, there may be biases in quantifying frailty. Then, our study used interrupted time series, but only used two changes before and after. While this analysis can assess a change in outcome level, it cannot independently evaluate changes in pre-existing trends, which is a standard strength of multi-point ITSA. Finally, this article mainly targets the physical examination population, and further verification is still needed among the large-sample population.

## Conclusion

Frailty will increase the risk of acute infection, and people who have not recovered for at least 4 months after the widespread infection, especially females and older population. In the face of a long-term pandemic, people need to increase their own health management, with good mood, appropriate physical activities, sufficient intake of grains and vegetables, and reduced intake of sugary drinks playing a positive role in improving frailty and repeated infection, as well as increasing health benefits.

## Data Availability

The original contributions presented in the study are included in the article/Supplementary material, further inquiries can be directed to the corresponding author.
