# Supplementary material for: The problem of frailty caused by acute infection and future health management strategies to improve frailty
Source: Front Public Health. 2026 Jan 20;13:1735577. doi: 10.3389/fpubh.2025.1735577 (PMC12864481; doi:10.3389/fpubh.2025.1735577)
Supplement: Supplementary file 1 [file Data_Sheet_1.docx]

**Additional file 1**

**The problem of frailty caused by acute infection and future health management strategies to improve frailty**

Guihua Li^1,¶^,Yue Zhao^1,¶^, Wenhui Gu^1^, Qianqian Wang^1^, Xinyi Lu^2^, Xinlei Miao^1, 3,*^

^1^ Health Management Center, The Second hospital of Dalian Medical University, 116023, Dalian, Liaoning, China;

^2^ Peking University Third Hospital, 100191, Beijing, China;

^3^ Research Center, The Second hospital of Dalian Medical University, 116023, Dalian, Liaoning, China;

^4^ Beijing Municipal Key Laboratory of Clinical Epidemiology, School of Public Health, Capital Medical University, Beijing, China.

**Contents:**

***Fig S1*** *Flow chart.................................................................................3*

***Table S1*** *Items included in the frailty index...............................................4*

***Cocariates definition****.................................................................................5*

***Fig S2*** *Kaplan-Meier estimates of the uninfected rate of different frailty status and the parallelism test.........................................................7*

***Table S2*** *The changes in frailty index between infected and uninfected individuals. ...............................................................................................8*

***Fig S3*** *Trends in biomarker levels before and after acute infection in male.........................................................................................10*

***Fig S4*** *Trends in biomarker levels before and after acute infection in female......................................................................................11*

***Fig S5*** *Trends in biomarker levels before and after acute infection in the age groups of 20-44.........................................................12*

***Table S3*** *Model estimates of level and trend change using interrupted time series analyses for the primary outcomes, overall and by sex.........14*

***Fig S6*** *Trends in biomarker levels before and after acute infection in the age groups of 45-64.........................................................21*

***Fig S7*** *Trends in biomarker levels before and after acute infection in the age groups of 65 and above.............................................23*

***Fig S8*** *Trends in biomarker levels before and after acute infection in the age groups of 65 and above.............................................25*

***Table S4*** *Model estimates of level and trend change using interrupted time series analyses for the primary outcomes by age. ...........................26*

***Table S5*** *Model estimates of level and trend change using interrupted time series analyses for the infected and uninfected people.....................34*

***Table S6*** *Model estimates of level and trend change using interrupted time series analyses for the infected and uninfected................................40*


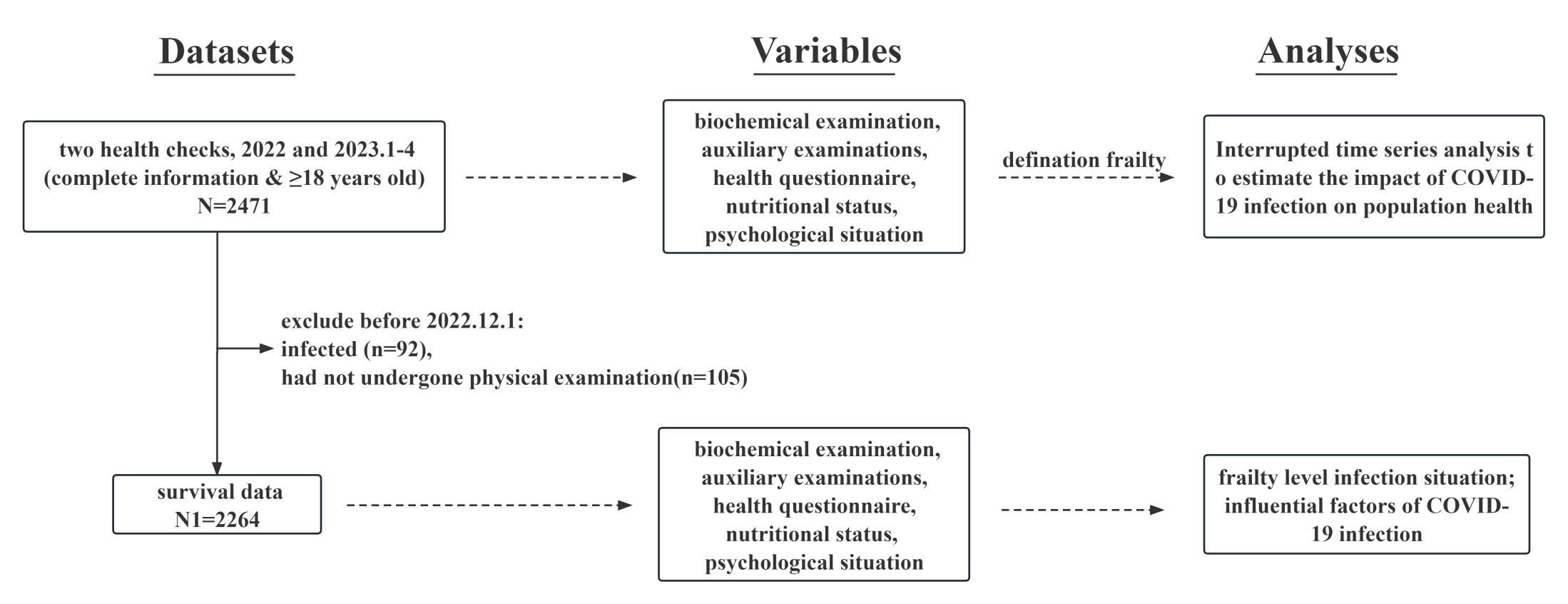
**Fig S1** Flow chart

**Table S1** Items included in the frailty index

| Items | Variables | Data Type | Cutoff point |
| --- | --- | --- | --- |
| 1.Blood routine test | Red blood cell | Binary | >=3.5*10^12/L & <=5*10^12/L = 0 & sex=female ;>=4.0*10^12/L & <=5.5*10^12/L = 0 & sex=male ; else = 1 |
|  | White blood cell | Binary | >=4*10^9/L & <=10*10^9/L= 0 ;else = 1 |
|  | Lymphocyte (n and %) | Binary | >=20% & <=40%= 0 ;else = 1 |
|  | Monocyte (n and %) | Binary | >=3% & <=8%= 0 ;else = 1 |
|  | Neutrophil (n and %) | Binary | >=40% & <=75%= 0 ;else = 1 |
| 2.Kidney function | Serum uric acid | Binary | >=89μmol/L & <=357μmol/L = 0 & sex=female ;>=150μmol/L & <=416μmol/L = 0 & sex=male ; else = 1 |
|  | Urea | Binary | >=3.2mmoL& <=7.1mmoL= 0 ;else = 1 |
|  | Creatinine | Binary | >=44μmol/L & <=97μmol/L = 0 & sex=female ;>=53μmol/L & <=106μmol/L = 0 & sex=male ; else = 1 |
| 3.Liver function | Total bilirubin | Binary | >=3.4μmol/L & <=17.1μmol/L = 0;else = 1 |
|  | Lactate dehydrogenase | Binary | >=120 U/L & <=250U/L = 0;else = 1 |
|  | Aspartate aminotransferase/Alanine aminotransferase | Binary | if value=1.15, defined=0; else=1 |
|  | Aspartate aminotransferase | Binary | >=8 U/L & <=40U/L = 0;else = 1 |
|  | Alanine aminotransferase | Binary | >=5 U/L & <=40U/L = 0;else = 1 |
|  | Alkaline phosphatase | Binary | >=30 U/L & <=100U/L = 0 & sex=female & 20≤age≤49 ;>=50U/L & <=135U/L = 0 & sex=female & 50≤age≤79 ;>=45 U/L & <=125U/L = 0 & sex=male ; else = 1 |
|  | γ-glutamyl transferase | Binary | >=7U/L & <=32U/L = 0 & sex=female ;>=11U/L & <=50U/L = 0 & sex=male ; else = 1 |
| 4.Thyroid function | Sensitive thyroid stimulating hormone | Binary | >=2mU/L & <=10mU/L = 0;else = 1 |
|  | Free triiodothyronine | Binary | >=6.0pmoL& <=11.4pmoL= 0 ;else = 1 |
|  | Free thyroxine | Binary | >=10.3pmoL& <=25.7pmoL= 0 ;else = 1 |
|  | Thyroglobulin antigen | Binary | >=3ng/mL & <=40ng/mL = 0;else = 1 |
|  | Thyroid peroxidase antibodies | Binary | >=0IU/mL & <=43IU/mL = 0;else = 1 |
| 5.Tumor markers | Alphafetoprotein | Binary | <25μg/L (RIA,CLIA,ELISA)= 0 ;else = 1 |
|  | Carcinoembryonic antigen | Binary | <5μg/L (RIA,CLIA,ELISA)= 0 ;else = 1 |
|  | carbohydrate antigen 199 | Binary | <3.7万U/L(CLIA,RIA,ELISA) = 0 ;else = 1 |
|  | Cancer antigen 242 | Binary | <20kU/L(ELISA) = 0 ;else = 1 |
|  | Cancer antigen 153 | Binary | <2.5万U/L(CLIA,RIA,ELISA) = 0 ;else = 1 |
|  | Serum ferritin | Binary | >=12μg/L & <=150μg/L = 0 & sex=female ;>=15μg/L & <=200μg/L = 0 & sex=male ; else = 1 |
|  | Human growth hormone | Binary | <10μg/L = 0 & sex=female ;<2μg/L = 0 & sex=male ; else = 1 |
|  | Free prostate-specific antigen | Binary | <0.8μg/L (RIA,CLIA,ELISA)= 0 ;else = 1 |
|  | β-human chorionic gonadotropin | Binary | <5U/L= 0 ;else = 1 |
| 6.Erythrocyte sedimentation rate |  | Binary | >=0mm/1h & <=20mm/1h = 0 & sex=female ;>=0mm/1h & <=15mm/1h = 0 & sex=male ; else = 1 |
| 7.Troponin I |  | Binary | >1.5μg/L/<0.2μg/L = 0;else = 1 |
| 8.Vitamin D |  | Binary | >=20ng/mL & <=50ng/mL = 0;else = 1 |
| 9.14C-Urea breath test |  | Binary | Participants with a negative ¹⁴C-urea breath test result were assigned a value of 0, while those with a positive result were assigned a value of 1. |
| 10.Physical measures | Diastolic blood pressure | Binary | <=89mmHg=0;else = 1 |
|  | Systolic blood pressure | Binary | <=139mmHg=0;else = 1 |
|  | Abdominal circumference | Binary | <=85cm= 0 & sex=female ;<=90cm= 0 & sex=male ; else = 1 |
|  | Body mass index | Binary | >=18.5kg/㎡& <=24.9kg/㎡= 0 ;else = 1 |
|  | Heart rate | Binary | >=60次/分& <=100次/分= 0 ;else = 1 |
| 11.Blood glucose | Glycosylated hemoglobin | Binary | >=4 mmol/mol & <=6 mmol/mol = 0 ;else = 1 |
|  | Fasting blood glucose | Binary | >=3.9 mmol/L & <=6.1 mmol/L = 0 ;else = 1 |
| 12.Serum lipid | Triglyceride | Binary | >=0.56 mmol/L & <=1.7mmol/L = 0 ;else = 1 |
|  | Total cholesterol | Binary | <=5.2 mmol/L = 0 ;>5.2 mmol/L = 1 |
|  | High-density lipoprotein cholesterol | Binary | >=1.03 mmol/L & <=2.07 mmol/L = 0 ;else = 1 |
|  | Low-density lipoprotein cholesterol | Binary | <=3.4 mmol/L = 0 ;else = 1 |
| 13.Image examinations | Fatty liver disease | Binary | Participants diagnosed by a physician as having no liver disease were designated as 0, and all others were assigned a value of 1. |
|  | Thyroid nodules | Binary | Participants with thyroid ultrasound findings showing no nodules were categorized as 0, while those with any nodules were assigned a value of 1. |
|  | Breast nodules | Binary | Participants with breast ultrasound findings showing no nodules were assigned a value of 0, and those with any nodule(s) present were assigned a value of 1. |
|  | Pulmonary nodules | Binary | Participants with lung CT scans showing no pulmonary nodules were assigned a value of 0, while all others (i.e., those with nodules present) were assigned a value of 1. |
|  | Cervical vascular abnormalities | Binary | Participants with normal carotid ultrasound findings were categorized as 0, while those with any abnormality were assigned a value of 1. |
|  | Lung inflammation | Binary | Participants with lung CT scans showing no inflammation were scored as 0; all others were scored as 1. |
| 14.History | Personal medicine history | Binary | Respondents reporting no medicine history were categorized as healthy |
|  | Personal medical history | Binary | Respondents reporting no chronic disease were categorized as healthy |
|  | Family medical history | Binary | Respondents reporting no family medicine history were categorized as healthy |

In our study, personal medical history was defined based on participant self-report of having ever received a physician’s diagnosis for any chronic disease (e.g., hypertension, diabetes, cardiovascular disease, chronic respiratory disease, etc.). We did not differentiate between specific diseases for the purpose of the frailty index construction; instead, we recorded a binary (0/1) variable indicating the presence (1) or absence (0) of any such diagnosed condition. Similarly, personal medication history was defined as the self-reported regular use of any prescribed medication for a chronic condition, also scored in a binary manner (0 = no regular medication use, 1 = regular use of one or more prescribed medications). Family history was assessed through participant self-report of whether any first-degree relative had been diagnosed by a physician with a chronic disease (such as cardiovascular disease, diabetes, or cancer). This was also recorded as a binary variable (0 = no, 1 = yes).

**Covariates Definition**

All variables were defined as binary classification variables. Smoking and drinking was categorized as yes (having related experience) or no (not having related experience). Metabolic equivalent (MET) was calculated from the relevant physical activity types and time reported by participants, and MET≤3 was defined as not having physical activity.^[1]^ We defined individuals under the age of 60 who sleep for more than 7 hours and those over 60 years who sleep for between 5 and 8 hours as normal sleep.^[2]^ Having good tooth brushing habits means brushing teeth twice or more a day. The dietary nutrition standards is evaluated according to the provisions of the Chinese Dietary Guidelines 2022.^[3]^ The evaluation criteria are the same as the rules for calculating FI, and ultimately the population who meets dietary standards is considered to have a dietary score of 0. The drinking amount of tea is defined as 2-3cups per day as mild intake, 4-5 cups as moderate intake, 6-8 cups as excessive intake, and more than 8 cups as severe intake based on the reference of less than one cup (250ml) per day. The soda water defined none intake per week, 1-5 cups per week, 6-9 cups per week, more than 10 cups per week as reference, mild intake, moderate intake, excessive intake, respectively. Compared to not drinking coffee, we defined 1-2 cups per week as mild intake, 3-5cups per week as moderate intake, and more than 5 cups as excessive intake. Mild intake of sugary drinks were defined as 1-2 bottles per week, 3-4 bottles of sugary drinks as moderate intake, and more than 4 bottles per week as excessive intake. There were 11 items about psychological stress for the subjects, and those with a total score of 0 were defined as optimistic about psychological stress. We defined nodules of BI-RADS 3 and above in thyroid ultrasound and C-TI-RADS 3 and above in breast ultrasound as having nodules. Ultrasound diagnosis of any degree of fatty liver was defined as having fatty liver. Pulmonary nodules and lung inflammation was defined through the diagnose of Low-dose computed tomography (LDCT). Cervical vascular abnormalities are defined as the phenomenon of plaques, narrowing, and thickening of the intima media in cervical blood vessels. The body mass index (BMI) was calculated by the ratio of weight (kg) and the square of height (m^2^), and BMI between 18.5 kg/m^2^ and 24 kg/m^2^ as a normal person. The definition of having a family history, personal history, and medication history was that the participant has any related historical event.

Kaplan-Meier estimates of the uninfected rate of different frailty status was shown in **Fig S2-A**. A total 1330 participants had an acute infection , and the median protection rate (uninfected rate) for mild frailty, moderate frailty and severe frailty participants were 26 days, 24 days and 27 days, respectively. The parallelism test shows that there was no statistically significant difference ($\chi^{2}=1.52, P=0.4682$) in protected rates among participants with different status of frailty (**Fig S2-B**).

The proportional risk hypothesis found that participants in different sex groups had different infection status over time. We correct sex by establishing an interaction term between sex and time to meet the modeling requirements of Cox model, we can see detail in **Fig S2-C** and **Fig S2-D**.


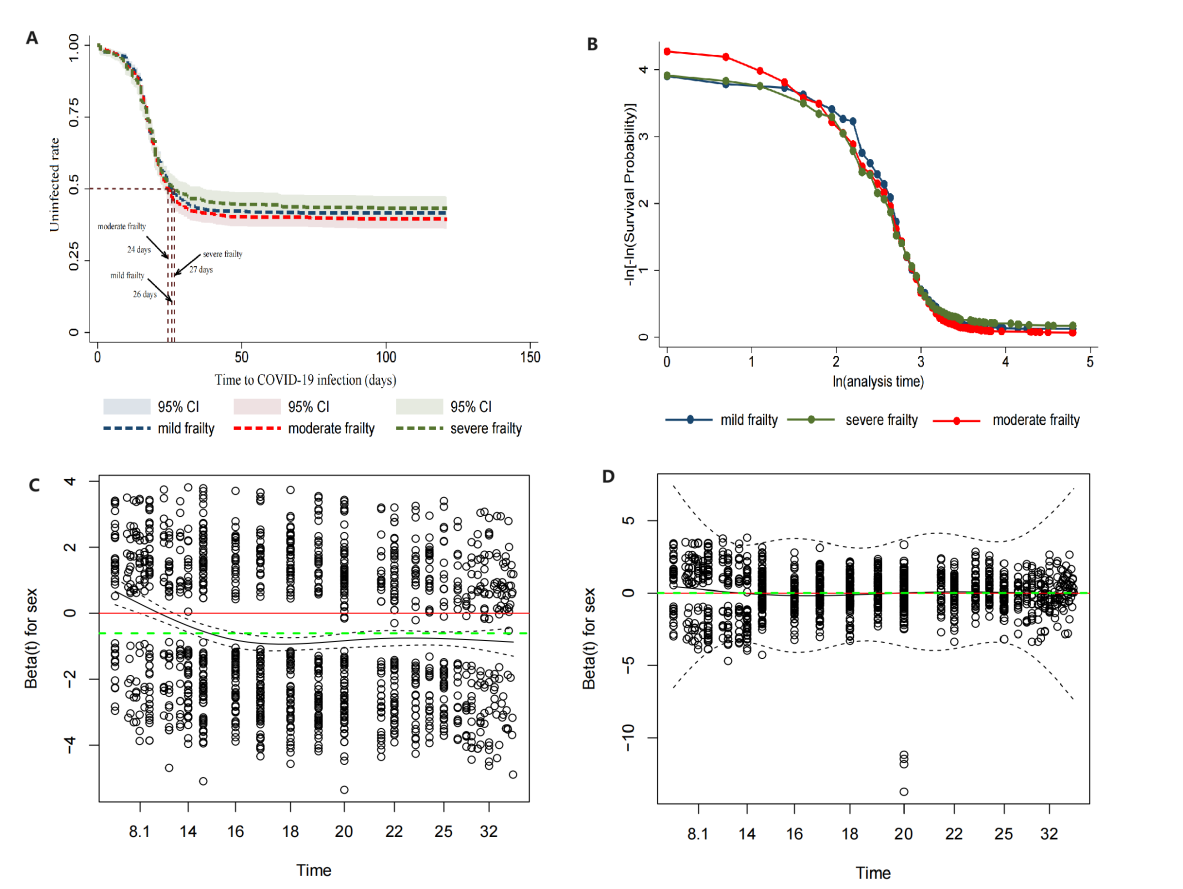


**Fig S2** Kaplan-Meier estimates of the uninfected rate of different frailty status and the parallelism test

A shows the Kaplan-Meier estimates of the uninfected rate of different frailty status; B shows the parallelism test of the Cox model. C shows the sex variable in model failed the assumptions for a Cox PH model (p< 0.05). The black line means the coefficient of the sex and two dashed lines show the 95% confidence interval. The red line is the reference line. The green dashed line indicates that if the sex variable conforms to the risk assumption. D means the situation where the sex variable satisfies the proportional risk assumption after time interaction.

**Table S2** shows the changes in frailty index ($\beta_{2}$) between infected and uninfected individuals. Female (0.05264 [0.03357,0.07170], P<0.05) has greater change of FI than male (0.03283 [0.00974,0.05593, P<0.05]) among the infected people. FI among people aged over 65 years old (0.00699 [0.00305,0.01093], P<0.05) has been continuously significant increasing after infected four months.

Table S2 The changes in frailty index between infected and uninfected individuals

|  | Uninfected | Infected |
| --- | --- | --- |
| Total |  |  |
| $\beta_{0}$ | 0.36477(0.35267,0.37686)* | 0.35487(0.34525,0.36448)* |
| $\beta_{1}$ | -0.00006(-0.00008,-0.00003)* | -0.00002(-0.00003,-0.00001)* |
| $\beta_{2}$ | 0.03126(0.01072,0.05179)* | 0.02779(0.01276,0.04281)* |
| $\beta_{3}$ | 0.00014(0.00010,0.00017)* | 0.00006(0.00004,0.00008)* |
| Male |  |  |
| $\beta_{0}$ | 0.35684(0.34355,0.37014)* | 0.37749(0.36353,0.39144)* |
| $\beta_{1}$ | 0.00001(-0.00002,0.00005) | -0.00001(-0.00004,0.00003) |
| $\beta_{2}$ | 0.03264(0.00846,0.05683)* | 0.03283(0.00974,0.05593)* |
| $\beta_{3}$ | 0.00002(-0.00003,0.00008) | 0.00005(0.00001,0.00010)* |
| Female |  |  |
| $\beta_{0}$ | 0.32390(0.30320,0.34461)* | 0.31719(0.30535,0.32903)* |
| $\beta_{1}$ | -0.00028(-0.00041,-0.00014)* | -0.00003(-0.00006,-0.00001)* |
| $\beta_{2}$ | 0.04919(0.01431,0.08407)* | 0.05264(0.03357,0.07170)* |
| $\beta_{3}$ | 0.00043(0.00021,0.00064)* | 0.00003(-0.00001,0.00007) |
| 20-44 |  |  |
| $\beta_{0}$ | 0.32707(0.30975,0.34439)* | 0.32989(0.31834,0.34143)* |
| $\beta_{1}$ | 0.00001(-0.00007,0.00009) | -0.00002(-0.00004,0.00001) |
| $\beta_{2}$ | 0.02962(0.00326,0.05597)* | 0.04055(0.02320,0.05790)* |
| $\beta_{3}$ | 0.00004(0.00001,0.00007)* | 0.00004(0.00001,0.00007) |
| 45-65 |  |  |
| $\beta_{0}$ | 0.37404(0.35644,0.39164)* | 0.39123(0.37600,0.40645)* |
| $\beta_{1}$ | -0.00008(-0.00001,-0.00009)* | -0.00008(-0.00001,-0.00004)* |
| $\beta_{2}$ | 0.033331(0.00430,0.06233)* | 0.02364(-0.00282,0.05011) |
| $\beta_{3}$ | 0.00022(0.00011,0.00032)* | 0.00023(0.00016,0.00030)* |
| 65+ |  |  |
| $\beta_{0}$ | 0.37540(0.33008,0.42071)* | 0.35788(0.27760,0.43815)* |
| $\beta_{1}$ | -0.00288(-0.00382,-0.00194)* | -0.00368(-0.00665,-0.00070)* |
| $\beta_{2}$ | 0.08538(0.02624,0.14451)* | 0.01935(-0.05749,0.09619) |
| $\beta_{3}$ | 0.00386(0.00229,0.00542)* | 0.00699(0.00305,0.01093)* |

Note. * means P<0.05;$\beta_{0}$ estimates the frailty index at the beginning of the time series. $\beta_{1}$ estimates the preinfection trend of frailty index. $\beta_{2}$ estimates the immediate change in the level of frailty indexafter infection. $\beta_{3}$ estimates the after infection trend of frailty index.

Fig S3 revealed that 20 biomarkers in the total population had a significant immediate change after infection (SBP, heart rate, AC, white blood cell, red blood cell, lymphocyte, HbA1c, triglyceride, HDL-C, lactate dehydrogenase, AST, alkaline phosphatase, serum uric acid, urine creatinine, FT4, 14C-Urea breath tests, ESR, CA153, CA199, free prostate specific antigen, P<0.05), and 25 biomarkers had a long-term abnormal trend significantly (SBP, DBP, heart rate, AC, white blood cell, red blood cell, neutrophil%, neutrophil, lymphocyte, monocyte%, monocyte, glucose, triglyceride, HDL-C, AST/ALT, AST, ALT, alkaline phosphatase, $\gamma$-GGT, serum uric acid, urea, urine creatinine, FT4, vitamin D, CEA, P<0.05).


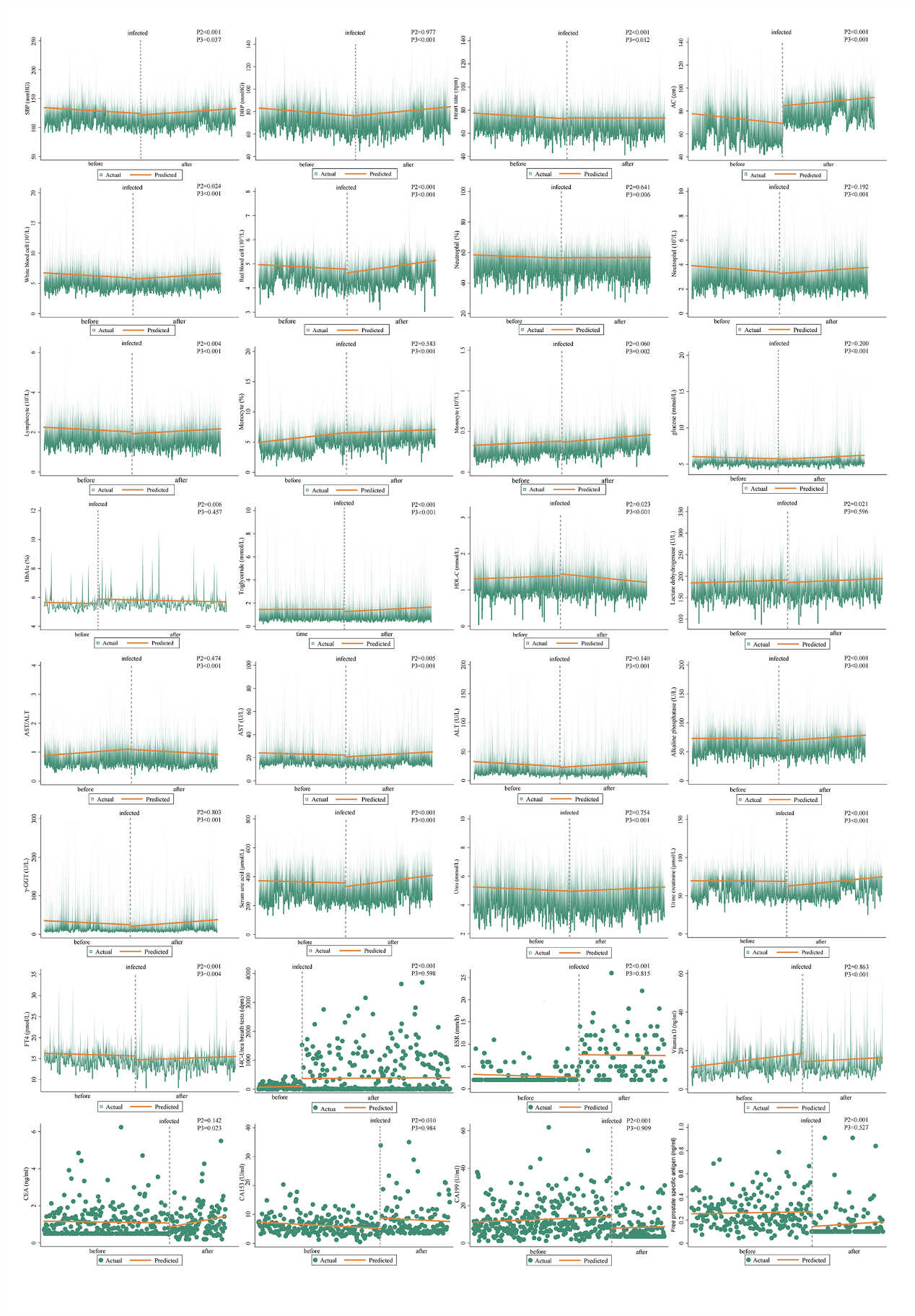


Fig S3 Changes in blood biomarkers caused by acute infection in total population.

P2 and P3 in the Fig represent the corresponding P values of β_2_ and β_3_ in interrupted time series analysis, respectively. β_2_ and β_3_ estimate the immediate change and trend in the level of biomarkers after infection, respectively. Green lines and dots represent the actual values of biomarkers, gray dotted lines represent infection, and orange solid lines represent fitting lines for the average value of biomarkers.

*Abbreviations*: *SBP* systolic blood pressure, *DBP* diastolic blood pressure, *BMI* body mass index, *AC* abdominal circumference, *LDL-C* low density lipoprotein cholesterol, *AST/ALT* aspartate aminotransferase/alanine aminotransferase, *ALT* alanine aminotransferase, *γ-GGT* gamma-glutamyl transpeptidase, *anti tg* thyroglobulin antibody.

**Fig S4** revealed that 10 biomarkers in the male population had a significant immediate change after infection (DBP, BMI, AC, monocyte%, monocyte, total cholesterol, LDL-C, lactate dehydrogenase, 14C-Urea breath tests, vitamin D , P<0.05), and 16 biomarkers had a long-term abnormal trend significantly (SBP, DBP, heart rate, AC, white blood cell, neutrophil %, neutrophil, lymphocyte, monocyte%, glucose, total bilirubin, AST/ALT, ALT, γ-GGT, anti tg, 14C-Urea breath tests, P<0.05).


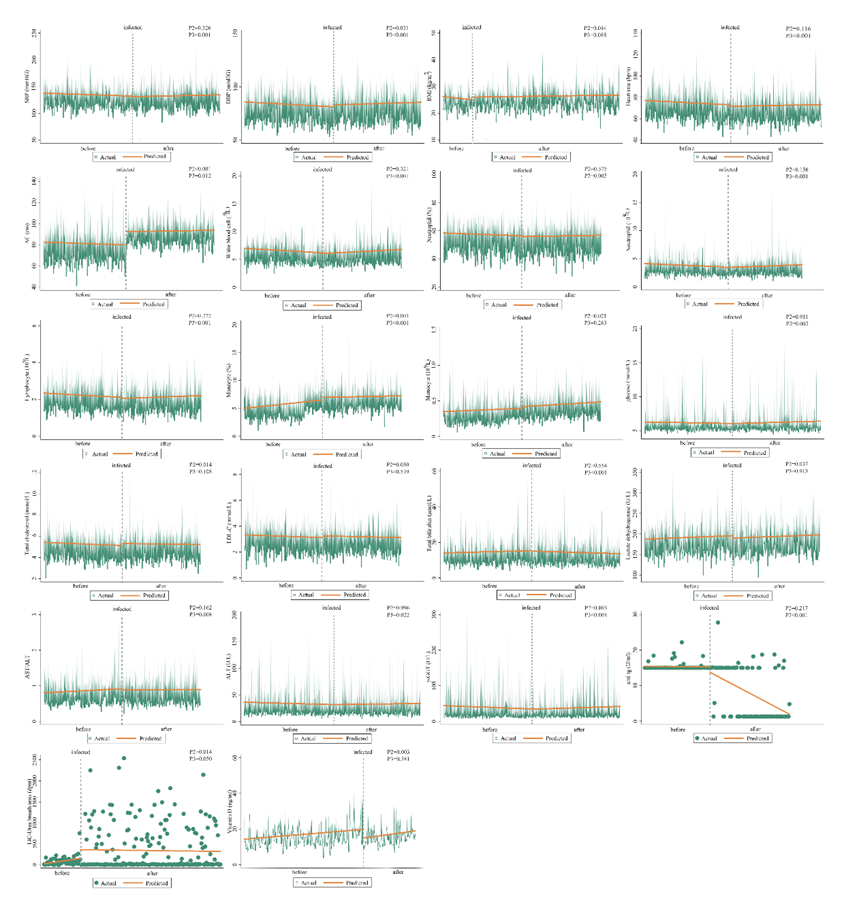


Fig S4 Trends in biomarker levels before and after acute infection in male.

P2 and P3 in the Fig represent the corresponding P values of β_2_ and β_3_ in interrupted time series analysis, respectively. β_2_ and β_3_ estimate the immediate change and trend in the level of biomarkers after infection, respectively. Green lines and dots represent the actual values of biomarkers, gray dotted lines represent infection, and orange solid lines represent fitting lines for the average value of biomarkers.

*Abbreviations*: *SBP* systolic blood pressure, *DBP* diastolic blood pressure, *BMI* body mass index, *AC* abdominal circumference, *LDL-C* low density lipoprotein cholesterol, *AST/ALT* aspartate aminotransferase/alanine aminotransferase, *ALT* alanine aminotransferase, *γ-GGT* gamma-glutamyl transpeptidase, *anti tg* thyroglobulin antibody.

**Fig S5** revealed that 16 biomarkers in the male population had a significant immediate change after infection (heart rate, AC, HbA1c, triglyceride, AST/ALT, ALT, serum uric acid, urine creatinine, FT4, 14C-Urea breath tests, ESR, vitamin D, CA153, CA199, CA242, βHCG , P<0.05), and 15 biomarkers had a long-term abnormal trend significantly (SBP, DBP, heart rate, white blood cell, neutrophil %, neutrophil, monocyte%, monocyte, triglyceride, total cholesterol, LDL-C, alkaline phosphatase, urine creatinine, FT4, vitamin D, P<0.05).


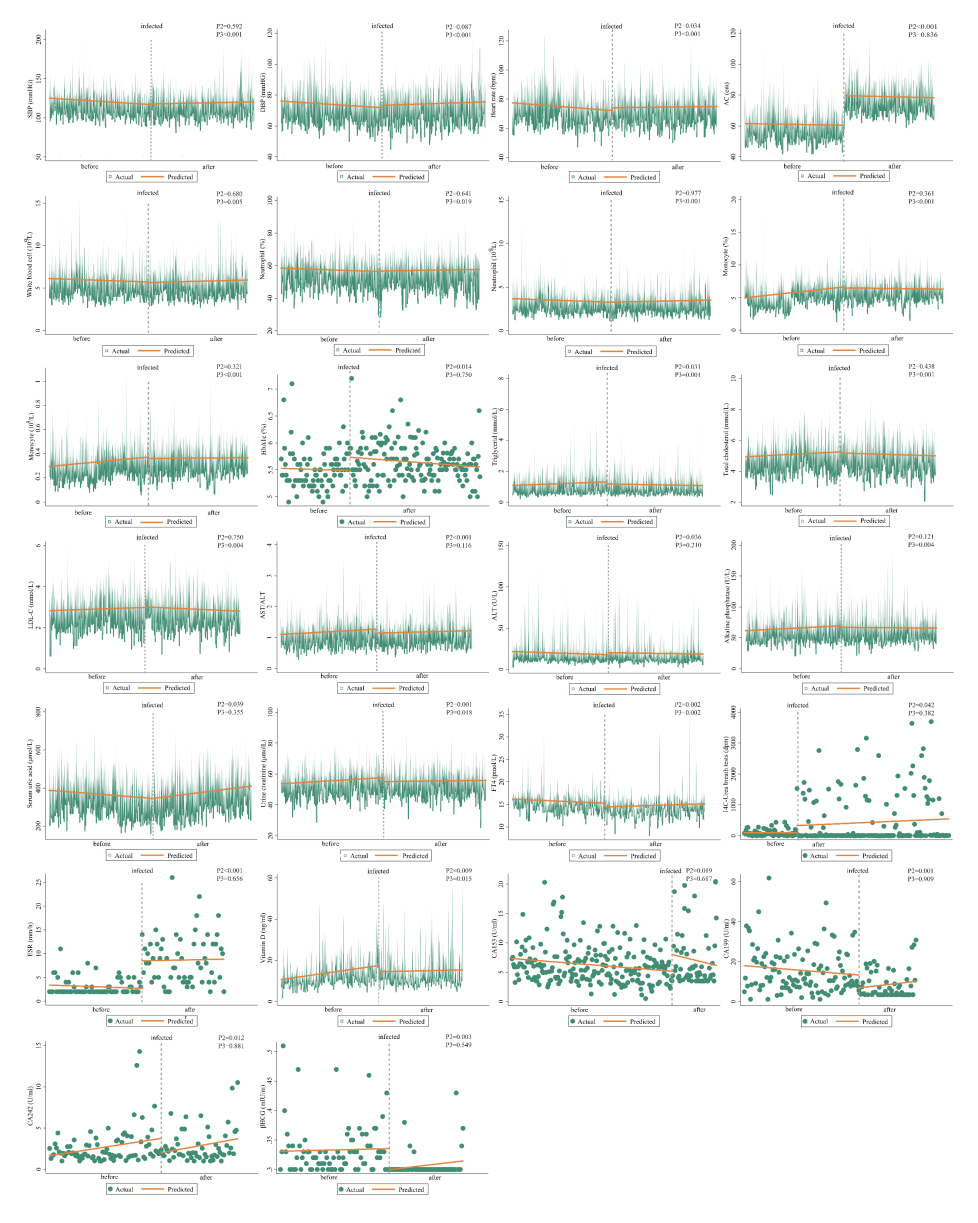


Fig S5 Trends in biomarker levels before and after acute infection in female.

P2 and P3 in the Fig represent the corresponding P values of β_2_ and β_3_ in interrupted time series analysis, respectively. β_2_ and β_3_ estimate the immediate change and trend in the level of biomarkers after infection, respectively. Green lines and dots represent the actual values of biomarkers, gray dotted lines represent infection, and orange solid lines represent fitting lines for the average value of biomarkers.

*Abbreviations*: *SBP* systolic blood pressure, *DBP* diastolic blood pressure, *AC* abdominal circumference, *HbA1c* glycosylated hemoglobin, *LDL-C* low density lipoprotein cholesterol, *AST/ALT* aspartate aminotransferase/alanine aminotransferase, *ALT* alanine aminotransferase, *FT4* free thyroxine, *ESR* erythrocyte sedimentation rate, *CA153* cancer antigen 153, *CA199* cancer antigen 199, *CA242* cancer antigen 242, *βHCG* β-human chorionic gonadotropin.

There were 13, 22 and 18 biomarkers have no significant statistical significance in the immediate changes before and after infection and the long-term effects after infection in total population, male and female, respectively. (**Table S3**)

**Table S3** Model estimates of level and trend change using interrupted time series analyses for the primary outcomes, overall and by sex

| Variables | β | Total | | | | | |  | Male | | | | | |  | Female | | | | | |
| --- | --- | --- | --- | --- | --- | --- | --- | --- | --- | --- | --- | --- | --- | --- | --- | --- | --- | --- | --- | --- | --- |
|  |  | Coef. | Std. Err. | t | P value | lower 95%CI | upper 95%CI |  | Coef. | Std. Err. | t | P value | lower 95%CI | upper 95%CI |  | Coef. | Std. Err. | t | P value | lower 95%CI | upper 95%CI |
| BMI  (kg/m^2^) | β0 | 23.68 | 0.34 | 69.69 | <0.001 | 23.01 | 24.34 |  | - | - | - | - | - | - |  | 22.34 | 0.31 | 72.41 | <0.001 | 21.73 | 22.94 |
|  | β1 | 0.00 | 0.00 | 0.21 | 0.83 | 0.00 | 0.00 |  | - | - | - | - | - | - |  | 0.00 | 0.00 | 0.82 | 0.41 | 0.00 | 0.00 |
|  | β2 | 0.18 | 0.33 | 0.55 | 0.58 | -0.46 | 0.83 |  | - | - | - | - | - | - |  | 0.09 | 0.39 | 0.24 | 0.81 | -0.67 | 0.85 |
|  | β3 | 0.00 | 0.00 | 1.13 | 0.26 | 0.00 | 0.00 |  | - | - | - | - | - | - |  | 0.00 | 0.00 | -0.25 | 0.80 | 0.00 | 0.00 |
| Red blood cell  (10^12^/L) | β0 | - | - | - | - | - | - |  | 5.17 | 0.02 | 254.65 | <0.001 | 5.13 | 5.21 |  | 4.49 | 0.02 | 217.70 | <0.001 | 4.45 | 4.53 |
|  | β1 | - | - | - | - | - | - |  | 0.00 | 0.00 | -1.21 | 0.23 | 0.00 | 0.00 |  | 0.00 | 0.00 | -0.02 | 0.98 | 0.00 | 0.00 |
|  | β2 | - | - | - | - | - | - |  | 0.03 | 0.03 | 1.02 | 0.31 | -0.03 | 0.08 |  | -0.05 | 0.03 | -1.73 | 0.08 | -0.11 | 0.01 |
|  | β3 | - | - | - | - | - | - |  | 0.00 | 0.00 | 1.64 | 0.10 | 0.00 | 0.00 |  | 0.00 | 0.00 | 1.51 | 0.13 | 0.00 | 0.00 |
| Lymphocyte  (%) | β0 | 34.37 | 0.33 | 104.38 | <0.001 | 33.73 | 35.02 |  | 34.31 | 0.41 | 84.28 | <0.001 | 33.51 | 35.11 |  | 2.05 | 0.03 | 58.49 | <0.001 | 1.98 | 2.12 |
|  | β1 | 0.00 | 0.00 | 0.47 | 0.64 | 0.00 | 0.00 |  | 0.00 | 0.00 | 0.16 | 0.87 | 0.00 | 0.00 |  | 0.00 | 0.00 | -1.44 | 0.15 | 0.00 | 0.00 |
|  | β2 | -0.38 | 0.45 | -0.85 | 0.40 | -1.25 | 0.50 |  | 0.01 | 0.59 | 0.01 | 0.99 | -1.15 | 1.16 |  | -0.06 | 0.05 | -1.17 | 0.24 | -0.15 | 0.04 |
|  | β3 | 0.00 | 0.00 | -1.39 | 0.17 | 0.00 | 0.00 |  | 0.00 | 0.00 | -1.40 | 0.16 | 0.00 | 0.00 |  | 0.00 | 0.00 | 1.57 | 0.12 | 0.00 | 0.00 |
| Lymphocyte  (10^9^/L) | β0 | - | - | - | - | - | - |  | - | - | - | - | - | - |  | 34.55 | 0.51 | 67.27 | <0.001 | 33.54 | 35.56 |
|  | β1 | - | - | - | - | - | - |  | - | - | - | - | - | - |  | 0.00 | 0.00 | 0.32 | 0.75 | 0.00 | 0.00 |
|  | β2 | - | - | - | - | - | - |  | - | - | - | - | - | - |  | -0.72 | 0.69 | -1.05 | 0.29 | -2.07 | 0.62 |
|  | β3 | - | - | - | - | - | - |  | - | - | - | - | - | - |  | 0.00 | 0.00 | -0.47 | 0.64 | 0.00 | 0.00 |
| FBG  (mmol/L) | β0 | - | - | - | - | - | - |  | - | - | - | - | - | - |  | 5.54 | 0.06 | 97.34 | <0.001 | 5.43 | 5.65 |
|  | β1 | - | - | - | - | - | - |  | - | - | - | - | - | - |  | 0.00 | 0.00 | -0.33 | 0.74 | 0.00 | 0.00 |
|  | β2 | - | - | - | - | - | - |  | - | - | - | - | - | - |  | 0.13 | 0.08 | 1.68 | 0.09 | -0.02 | 0.28 |
|  | β3 | - | - | - | - | - | - |  | - | - | - | - | - | - |  | 0.00 | 0.00 | -0.45 | 0.65 | 0.00 | 0.00 |
| HbA1c  (%) | β0 | - | - | - | - | - | - |  | 6.36 | 0.57 | 11.13 | <0.001 | 5.23 | 7.48 |  | - | - | - | - | - | - |
|  | β1 | - | - | - | - | - | - |  | -0.02 | 0.01 | -1.05 | 0.30 | -0.04 | 0.01 |  | - | - | - | - | - | - |
|  | β2 | - | - | - | - | - | - |  | 0.72 | 0.39 | 1.86 | 0.06 | -0.04 | 1.49 |  | - | - | - | - | - | - |
|  | β3 | - | - | - | - | - | - |  | 0.01 | 0.01 | 0.89 | 0.38 | -0.02 | 0.04 |  | - | - | - | - | - | - |
| Triglyceride  (mmol/L) | β0 | - | - | - | - | - | - |  | 1.69 | 0.09 | 19.57 | <0.001 | 1.52 | 1.86 |  | - | - | - | - | - | - |
|  | β1 | - | - | - | - | - | - |  | 0.00 | 0.00 | 0.43 | 0.67 | 0.00 | 0.00 |  | - | - | - | - | - | - |
|  | β2 | - | - | - | - | - | - |  | -0.07 | 0.11 | -0.61 | 0.54 | -0.28 | 0.14 |  | - | - | - | - | - | - |
|  | β3 | - | - | - | - | - | - |  | 0.00 | 0.00 | 0.28 | 0.78 | 0.00 | 0.00 |  | - | - | - | - | - | - |
| Total cholesterol  (mmol/L) | β0 | 5.22 | 0.04 | 129.27 | <0.001 | 5.14 | 5.30 |  | - | - | - | - | - | - |  | - | - | - | - | - | - |
|  | β1 | 0.00 | 0.00 | -0.63 | 0.53 | 0.00 | 0.00 |  | - | - | - | - | - | - |  | - | - | - | - | - | - |
|  | β2 | 0.03 | 0.06 | 0.43 | 0.66 | -0.09 | 0.14 |  | - | - | - | - | - | - |  | - | - | - | - | - | - |
|  | β3 | 0.00 | 0.00 | 0.33 | 0.74 | 0.00 | 0.00 |  | - | - | - | - | - | - |  | - | - | - | - | - | - |
| LDL-C  (mmol/L) | β0 | 3.14 | 0.03 | 90.78 | <0.001 | 3.07 | 3.21 |  | - | - | - | - | - | - |  | - | - | - | - | - | - |
|  | β1 | 0.00 | 0.00 | -1.62 | 0.11 | 0.00 | 0.00 |  | - | - | - | - | - | - |  | - | - | - | - | - | - |
|  | β2 | 0.02 | 0.05 | 0.33 | 0.74 | -0.08 | 0.11 |  | - | - | - | - | - | - |  | - | - | - | - | - | - |
|  | β3 | 0.00 | 0.00 | 1.43 | 0.15 | 0.00 | 0.00 |  | - | - | - | - | - | - |  | - | - | - | - | - | - |
| HDL-C  (mmol/L) | β0 | - | - | - | - | - | - |  | 1.22 | 0.02 | 68.91 | <0.001 | 1.19 | 1.26 |  | 1.51 | 0.02 | 61.74 | <0.001 | 1.46 | 1.55 |
|  | β1 | - | - | - | - | - | - |  | 0.00 | 0.00 | -0.84 | 0.40 | 0.00 | 0.00 |  | 0.00 | 0.00 | 1.34 | 0.18 | 0.00 | 0.00 |
|  | β2 | - | - | - | - | - | - |  | 0.00 | 0.02 | -0.14 | 0.89 | -0.05 | 0.04 |  | -0.03 | 0.04 | -0.89 | 0.37 | -0.10 | 0.04 |
|  | β3 | - | - | - | - | - | - |  | 0.00 | 0.00 | 0.08 | 0.94 | 0.00 | 0.00 |  | 0.00 | 0.00 | -1.19 | 0.23 | 0.00 | 0.00 |
| Total bilirubin  (μmol/L) | β0 | 13.90 | 0.23 | 60.12 | <0.001 | 13.44 | 14.35 |  | - | - | - | - | - | - |  | 12.97 | 0.34 | 38.36 | <0.001 | 12.31 | 13.64 |
|  | β1 | 0.00 | 0.00 | 0.03 | 0.98 | 0.00 | 0.00 |  | - | - | - | - | - | - |  | 0.00 | 0.00 | -0.97 | 0.33 | 0.00 | 0.00 |
|  | β2 | -0.53 | 0.32 | -1.64 | 0.10 | -1.17 | 0.10 |  | - | - | - | - | - | - |  | 0.06 | 0.44 | 0.13 | 0.90 | -0.80 | 0.92 |
|  | β3 | 0.00 | 0.00 | 0.65 | 0.51 | 0.00 | 0.00 |  | - | - | - | - | - | - |  | 0.00 | 0.00 | 0.34 | 0.74 | 0.00 | 0.00 |
| Lactate dehydrogenase (U/L) | β0 | - | - | - | - | - | - |  | 78.39 | 1.11 | 70.64 | <0.001 | 76.22 | 80.57 |  | 172.97 | 3.46 | 49.93 | <0.001 | 166.16 | 179.78 |
|  | β1 | - | - | - | - | - | - |  | 0.00 | 0.00 | 0.22 | 0.83 | 0.00 | 0.00 |  | 0.01 | 0.02 | 0.52 | 0.60 | -0.03 | 0.05 |
|  | β2 | - | - | - | - | - | - |  | -2.70 | 1.54 | -1.75 | 0.08 | -5.72 | 0.32 |  | -2.50 | 5.27 | -0.47 | 0.64 | -12.86 | 7.86 |
|  | β3 | - | - | - | - | - | - |  | 0.00 | 0.00 | 1.66 | 0.10 | 0.00 | 0.01 |  | 0.00 | 0.03 | 0.15 | 0.88 | -0.06 | 0.07 |
| AST  (U/L) | β0 | - | - | - | - | - | - |  | 26.30 | 0.69 | 38.17 | <0.001 | 24.94 | 27.65 |  | 20.82 | 0.52 | 40.34 | <0.001 | 19.81 | 21.84 |
|  | β1 | - | - | - | - | - | - |  | 0.00 | 0.00 | -0.78 | 0.44 | 0.00 | 0.00 |  | 0.00 | 0.00 | -1.28 | 0.20 | 0.00 | 0.00 |
|  | β2 | - | - | - | - | - | - |  | -0.95 | 0.82 | -1.16 | 0.25 | -2.55 | 0.66 |  | 0.35 | 0.74 | 0.47 | 0.64 | -1.11 | 1.80 |
|  | β3 | - | - | - | - | - | - |  | 0.00 | 0.00 | 1.87 | 0.06 | 0.00 | 0.00 |  | 0.00 | 0.00 | 0.88 | 0.38 | 0.00 | 0.00 |
| γ-GGT  (U/L) | β0 | - | - | - | - | - | - |  | - | - | - | - | - | - |  | 16.34 | 0.90 | 18.20 | <0.001 | 14.58 | 18.10 |
|  | β1 | - | - | - | - | - | - |  | - | - | - | - | - | - |  | 0.00 | 0.00 | 0.28 | 0.78 | 0.00 | 0.00 |
|  | β2 | - | - | - | - | - | - |  | - | - | - | - | - | - |  | 0.31 | 1.31 | 0.24 | 0.81 | -2.25 | 2.88 |
|  | β3 | - | - | - | - | - | - |  | - | - | - | - | - | - |  | 0.00 | 0.00 | -0.65 | 0.52 | -0.01 | 0.00 |
| Serum uric acid  (μmol/L) | β0 | - | - | - | - | - | - |  | 407.56 | 4.53 | 89.87 | <0.001 | 398.67 | 416.45 |  | - | - | - | - | - | - |
|  | β1 | - | - | - | - | - | - |  | 0.01 | 0.01 | 1.02 | 0.31 | -0.01 | 0.02 |  | - | - | - | - | - | - |
|  | β2 | - | - | - | - | - | - |  | 1.99 | 6.52 | 0.31 | 0.76 | -10.79 | 14.77 |  | - | - | - | - | - | - |
|  | β3 | - | - | - | - | - | - |  | -0.01 | 0.01 | -0.70 | 0.48 | -0.02 | 0.01 |  | - | - | - | - | - | - |
| Urea  (mmol/L) | β0 | - | - | - | - | - | - |  | 5.54 | 0.07 | 75.75 | <0.001 | 5.40 | 5.69 |  | 4.73 | 0.08 | 57.89 | <0.001 | 4.57 | 4.89 |
|  | β1 | - | - | - | - | - | - |  | 0.00 | 0.00 | -1.62 | 0.11 | 0.00 | 0.00 |  | 0.00 | 0.00 | -0.79 | 0.43 | 0.00 | 0.00 |
|  | β2 | - | - | - | - | - | - |  | 0.16 | 0.10 | 1.51 | 0.13 | -0.05 | 0.36 |  | 0.15 | 0.11 | 1.37 | 0.17 | -0.07 | 0.37 |
|  | β3 | - | - | - | - | - | - |  | 0.00 | 0.00 | 0.60 | 0.55 | 0.00 | 0.00 |  | 0.00 | 0.00 | -0.80 | 0.43 | 0.00 | 0.00 |
| Urine creatinine  (μmol/L) | β0 | - | - | - | - | - | - |  | 76.62 | 0.68 | 113.29 | <0.001 | 75.30 | 77.95 |  | - | - | - | - | - | - |
|  | β1 | - | - | - | - | - | - |  | 0.00 | 0.00 | 3.22 | 0.00 | 0.00 | 0.00 |  | - | - | - | - | - | - |
|  | β2 | - | - | - | - | - | - |  | -1.79 | 1.32 | -1.36 | 0.17 | -4.38 | 0.79 |  | - | - | - | - | - | - |
|  | β3 | - | - | - | - | - | - |  | 0.00 | 0.00 | -1.49 | 0.14 | -0.01 | 0.00 |  | - | - | - | - | - | - |
| FT4  (pmol/l) | β0 | - | - | - | - | - | - |  | 17.30 | 0.51 | 33.84 | <0.001 | 16.29 | 18.31 |  | - | - | - | - | - | - |
|  | β1 | - | - | - | - | - | - |  | 0.00 | 0.01 | 0.18 | 0.86 | -0.02 | 0.02 |  | - | - | - | - | - | - |
|  | β2 | - | - | - | - | - | - |  | -0.66 | 0.85 | -0.78 | 0.44 | -2.35 | 1.02 |  | - | - | - | - | - | - |
|  | β3 | - | - | - | - | - | - |  | 0.00 | 0.02 | -0.19 | 0.85 | -0.03 | 0.03 |  | - | - | - | - | - | - |
| STSH  (mIU/L) | β0 | 2.36 | 0.37 | 6.32 | <0.001 | 1.62 | 3.09 |  | 2.20 | 0.42 | 5.22 | <0.001 | 1.37 | 3.03 |  | 2.34 | 0.42 | 5.63 | <0.001 | 1.53 | 3.16 |
|  | β1 | 0.00 | 0.00 | 0.31 | 0.76 | 0.00 | 0.00 |  | 0.00 | 0.01 | -0.41 | 0.69 | -0.02 | 0.01 |  | 0.00 | 0.00 | 0.51 | 0.61 | 0.00 | 0.00 |
|  | β2 | -0.40 | 0.51 | -0.78 | 0.43 | -1.41 | 0.61 |  | 0.06 | 0.44 | 0.14 | 0.89 | -0.80 | 0.92 |  | -0.59 | 0.61 | -0.97 | 0.33 | -1.79 | 0.61 |
|  | β3 | 0.00 | 0.00 | 0.30 | 0.77 | 0.00 | 0.00 |  | 0.00 | 0.01 | 0.19 | 0.85 | -0.02 | 0.02 |  | 0.00 | 0.00 | 0.31 | 0.75 | 0.00 | 0.01 |
| anti tg  (U/ml) | β0 | 46.53 | 7.07 | 6.58 | <0.001 | 32.65 | 60.41 |  | - | - | - | - | - | - |  | 56.15 | 7.14 | 7.86 | <0.001 | 42.13 | 70.17 |
|  | β1 | -0.03 | 0.03 | -1.03 | 0.30 | -0.09 | 0.03 |  | - | - | - | - | - | - |  | -0.04 | 0.03 | -1.57 | 0.12 | -0.09 | 0.01 |
|  | β2 | 9.71 | 9.47 | 1.03 | 0.31 | -8.87 | 28.30 |  | - | - | - | - | - | - |  | 7.23 | 9.24 | 0.78 | 0.43 | -10.90 | 25.37 |
|  | β3 | -0.01 | 0.04 | -0.40 | 0.69 | -0.09 | 0.06 |  | - | - | - | - | - | - |  | 0.00 | 0.03 | -0.03 | 0.97 | -0.07 | 0.06 |
| anti tpo  (U/ml) | β0 | 190.83 | 37.37 | 5.11 | <0.001 | 117.48 | 264.17 |  | 73.90 | 24.61 | 3.00 | 0.00 | 25.32 | 122.49 |  | 226.24 | 37.47 | 6.04 | <0.001 | 152.70 | 299.79 |
|  | β1 | -0.19 | 0.15 | -1.24 | 0.22 | -0.49 | 0.11 |  | -0.12 | 0.26 | -0.46 | 0.65 | -0.63 | 0.40 |  | -0.26 | 0.14 | -1.87 | 0.06 | -0.53 | 0.01 |
|  | β2 | 41.43 | 45.93 | 0.90 | 0.37 | -48.73 | 131.58 |  | 80.82 | 72.95 | 1.11 | 0.27 | -63.22 | 224.87 |  | 39.54 | 43.71 | 0.90 | 0.37 | -46.24 | 125.33 |
|  | β3 | 0.18 | 0.20 | 0.92 | 0.36 | -0.21 | 0.58 |  | -0.48 | 1.29 | -0.37 | 0.71 | -3.03 | 2.06 |  | 0.30 | 0.18 | 1.66 | 0.10 | -0.05 | 0.65 |
| ESR  (mm/h) | β0 | - | - | - | - | - | - |  | 3.08 | 0.75 | 4.13 | <0.001 | 1.59 | 4.57 |  | - | - | - | - | - | - |
|  | β1 | - | - | - | - | - | - |  | -0.02 | 0.03 | -0.60 | 0.55 | -0.08 | 0.04 |  | - | - | - | - | - | - |
|  | β2 | - | - | - | - | - | - |  | 3.56 | 1.87 | 1.90 | 0.06 | -0.18 | 7.30 |  | - | - | - | - | - | - |
|  | β3 | - | - | - | - | - | - |  | -0.02 | 0.10 | -0.21 | 0.83 | -0.21 | 0.17 |  | - | - | - | - | - | - |
| AFP  (IU/ml) | β0 | 3.20 | 0.28 | 11.59 | <0.001 | 2.66 | 3.75 |  | 3.32 | 0.32 | 10.42 | <0.001 | 2.69 | 3.95 |  | 3.11 | 0.45 | 6.85 | <0.001 | 2.21 | 4.01 |
|  | β1 | 0.00 | 0.00 | -1.58 | 0.12 | -0.01 | 0.00 |  | -0.01 | 0.01 | -1.63 | 0.10 | -0.02 | 0.00 |  | -0.01 | 0.01 | -0.84 | 0.40 | -0.03 | 0.01 |
|  | β2 | 0.56 | 0.38 | 1.46 | 0.15 | -0.20 | 1.32 |  | 0.41 | 0.45 | 0.91 | 0.36 | -0.48 | 1.31 |  | 0.51 | 0.62 | 0.82 | 0.42 | -0.72 | 1.74 |
|  | β3 | 0.00 | 0.00 | 0.73 | 0.46 | 0.00 | 0.01 |  | 0.00 | 0.01 | 0.37 | 0.71 | -0.01 | 0.02 |  | 0.01 | 0.01 | 1.01 | 0.32 | -0.01 | 0.04 |
| CEA  (ng/ml) | β0 | - | - | - | - | - | - |  | 1.27 | 0.17 | 7.49 | <0.001 | 0.94 | 1.61 |  | 0.88 | 0.10 | 8.42 | <0.001 | 0.67 | 1.09 |
|  | β1 | - | - | - | - | - | - |  | 0.00 | 0.00 | 0.68 | 0.50 | 0.00 | 0.01 |  | 0.00 | 0.00 | 0.23 | 0.82 | 0.00 | 0.00 |
|  | β2 | - | - | - | - | - | - |  | -0.19 | 0.30 | -0.64 | 0.52 | -0.79 | 0.40 |  | -0.35 | 0.19 | -1.80 | 0.07 | -0.73 | 0.03 |
|  | β3 | - | - | - | - | - | - |  | 0.00 | 0.01 | 0.43 | 0.67 | -0.01 | 0.02 |  | 0.01 | 0.00 | 1.69 | 0.09 | 0.00 | 0.02 |
| CA199  (U/ml) | β0 | - | - | - | - | - | - |  | 9.85 | 1.03 | 9.57 | <0.001 | 7.82 | 11.88 |  | - | - | - | - | - | - |
|  | β1 | - | - | - | - | - | - |  | 0.01 | 0.01 | 1.13 | 0.26 | -0.01 | 0.03 |  | - | - | - | - | - | - |
|  | β2 | - | - | - | - | - | - |  | -2.90 | 2.11 | -1.37 | 0.17 | -7.06 | 1.27 |  | - | - | - | - | - | - |
|  | β3 | - | - | - | - | - | - |  | -0.04 | 0.05 | -0.72 | 0.47 | -0.14 | 0.07 |  | - | - | - | - | - | - |
| CA242  (U/ml) | β0 | 1.90 | 0.33 | 5.83 | <0.001 | 1.26 | 2.54 |  | 2.48 | 0.25 | 9.88 | <0.001 | 1.98 | 2.97 |  | - | - | - | - | - | - |
|  | β1 | 0.01 | 0.00 | 2.50 | 0.01 | 0.00 | 0.02 |  | 0.00 | 0.01 | 0.05 | 0.96 | -0.02 | 0.02 |  | - | - | - | - | - | - |
|  | β2 | -0.85 | 0.49 | -1.75 | 0.08 | -1.81 | 0.10 |  | 0.54 | 0.64 | 0.85 | 0.40 | -0.73 | 1.81 |  | - | - | - | - | - | - |
|  | β3 | 0.00 | 0.01 | -0.51 | 0.61 | -0.02 | 0.01 |  | -0.01 | 0.02 | -0.37 | 0.72 | -0.04 | 0.03 |  | - | - | - | - | - | - |
| Serum iron  (ng/ml) | β0 | 111.62 | 18.44 | 6.05 | <0.001 | 75.32 | 147.93 |  | 196.45 | 27.59 | 7.12 | <0.001 | 141.83 | 251.08 |  | 53.95 | 8.00 | 6.74 | <0.001 | 38.12 | 69.77 |
|  | β1 | -0.14 | 0.23 | -0.60 | 0.55 | -0.58 | 0.31 |  | -0.22 | 0.79 | -0.28 | 0.78 | -1.79 | 1.35 |  | -0.45 | 0.17 | -2.59 | 0.01 | -0.79 | -0.11 |
|  | β2 | 7.99 | 27.48 | 0.29 | 0.77 | -46.12 | 62.10 |  | 34.03 | 39.50 | 0.86 | 0.39 | -44.19 | 112.25 |  | 17.30 | 12.55 | 1.38 | 0.17 | -7.53 | 42.12 |
|  | β3 | 0.27 | 0.37 | 0.74 | 0.46 | -0.46 | 1.01 |  | -0.84 | 1.12 | -0.75 | 0.45 | -3.06 | 1.38 |  | 0.26 | 0.33 | 0.77 | 0.44 | -0.40 | 0.92 |
| βHCG  (mIU/m) | β0 | 0.34 | 0.01 | 28.46 | <0.001 | 0.31 | 0.36 |  | 0.34 | 0.02 | 16.33 | <0.001 | 0.30 | 0.38 |  | - | - | - | - | - | - |
|  | β1 | 0.00 | 0.00 | 0.50 | 0.61 | 0.00 | 0.00 |  | 0.00 | 0.00 | 0.80 | 0.43 | 0.00 | 0.00 |  | - | - | - | - | - | - |
|  | β2 | -0.03 | 0.02 | -1.86 | 0.06 | -0.07 | 0.00 |  | -0.02 | 0.03 | -0.80 | 0.43 | -0.08 | 0.04 |  | - | - | - | - | - | - |
|  | β3 | 0.00 | 0.00 | 0.36 | 0.72 | 0.00 | 0.00 |  | 0.00 | 0.00 | -0.61 | 0.54 | 0.00 | 0.00 |  | - | - | - | - | - | - |
| hGH  (ng/ml) | β0 | 0.82 | 0.09 | 8.96 | <0.001 | 0.64 | 1.00 |  | 0.56 | 0.06 | 8.96 | <0.001 | 0.43 | 0.68 |  | 0.99 | 0.15 | 6.47 | <0.001 | 0.69 | 1.29 |
|  | β1 | 0.00 | 0.00 | -1.02 | 0.31 | 0.00 | 0.00 |  | 0.00 | 0.00 | 0.29 | 0.77 | 0.00 | 0.00 |  | 0.00 | 0.00 | -0.99 | 0.33 | -0.01 | 0.00 |
|  | β2 | 0.11 | 0.14 | 0.82 | 0.41 | -0.16 | 0.38 |  | 0.04 | 0.09 | 0.40 | 0.69 | -0.14 | 0.21 |  | 0.10 | 0.24 | 0.42 | 0.67 | -0.37 | 0.58 |
|  | β3 | 0.00 | 0.00 | -0.34 | 0.74 | 0.00 | 0.00 |  | 0.00 | 0.00 | -1.05 | 0.30 | -0.01 | 0.00 |  | 0.00 | 0.01 | 0.20 | 0.84 | -0.01 | 0.01 |
| 1. PSA   (ng/ml) | β0 | - | - | - | - | - | - |  | 0.25 | 0.02 | 11.82 | <0.001 | 0.21 | 0.29 |  | - | - | - | - | - | - |
|  | β1 | - | - | - | - | - | - |  | 0.00 | 0.00 | 0.62 | 0.54 | 0.00 | 0.00 |  | - | - | - | - | - | - |
|  | β2 | - | - | - | - | - | - |  | -0.05 | 0.04 | -1.04 | 0.30 | -0.13 | 0.04 |  | - | - | - | - | - | - |
|  | β3 | - | - | - | - | - | - |  | 0.00 | 0.00 | -0.22 | 0.83 | 0.00 | 0.00 |  | - | - | - | - | - | - |

*Abbreviate*: *BMI* body mass index, *FBG* fasting blood glucose, *HbA1c* glycosylated hemoglobin, *LDL-C* low-density lipoprotein cholesterol, *HDL-C* high-density lipoprotein cholesterol, *AST* aspartate transaminase, *γ-GGT* γ-glutamyl transpeptidase, *FT4* free thyroxine, *sTSH* sensitive thyroid stimulating hormone, *anti tg* thyroglobulin antigen ,*anti tpo* thyroid peroxidase antibodies , *ESR* erythrocyte sedimentation rate, *AFP* alphafetoprotein, *CEA* carcinoembryonic antigen, *CA199* cancer antigen 199, *CA242* cancer antigen 242, *βHCG* β-human chorionic gonadotropin, *hGH* Human growth hormone, *F-PSA* fisher park summit alternative.

CI confidence interval, β0 constant term, β1 pre-intervention slope, β2 immediate effect, β3 long-term trend.

**Fig S6** revealed that 18 biomarkers in the age groups of 20-44 had a significant immediate change after infection (AC, white blood cell, red blood cell, lymphocyte, HbA1c, HDL-C, AST, ALT, alkaline phosphatase, γ-GGT, serum uric acid, urine creatinine, FT4, 14C-Urea breath tests, vitamin D, CA153, CA199, hGH, P<0.05), and 24 biomarkers had a long-term abnormal trend significantly (SBP, DBP, heart rate, AC, white blood cell, red blood cell, neutrophil, lymphocyte, monocyte%, glucose, triglyceride, LDL-C, HDL-C, AST/ALT, AST, ALT, alkaline phosphatase, γ-GGT, serum uric acid, urea, urine creatinine, FT4, AFP, free prostate specific antigen, P<0.05).
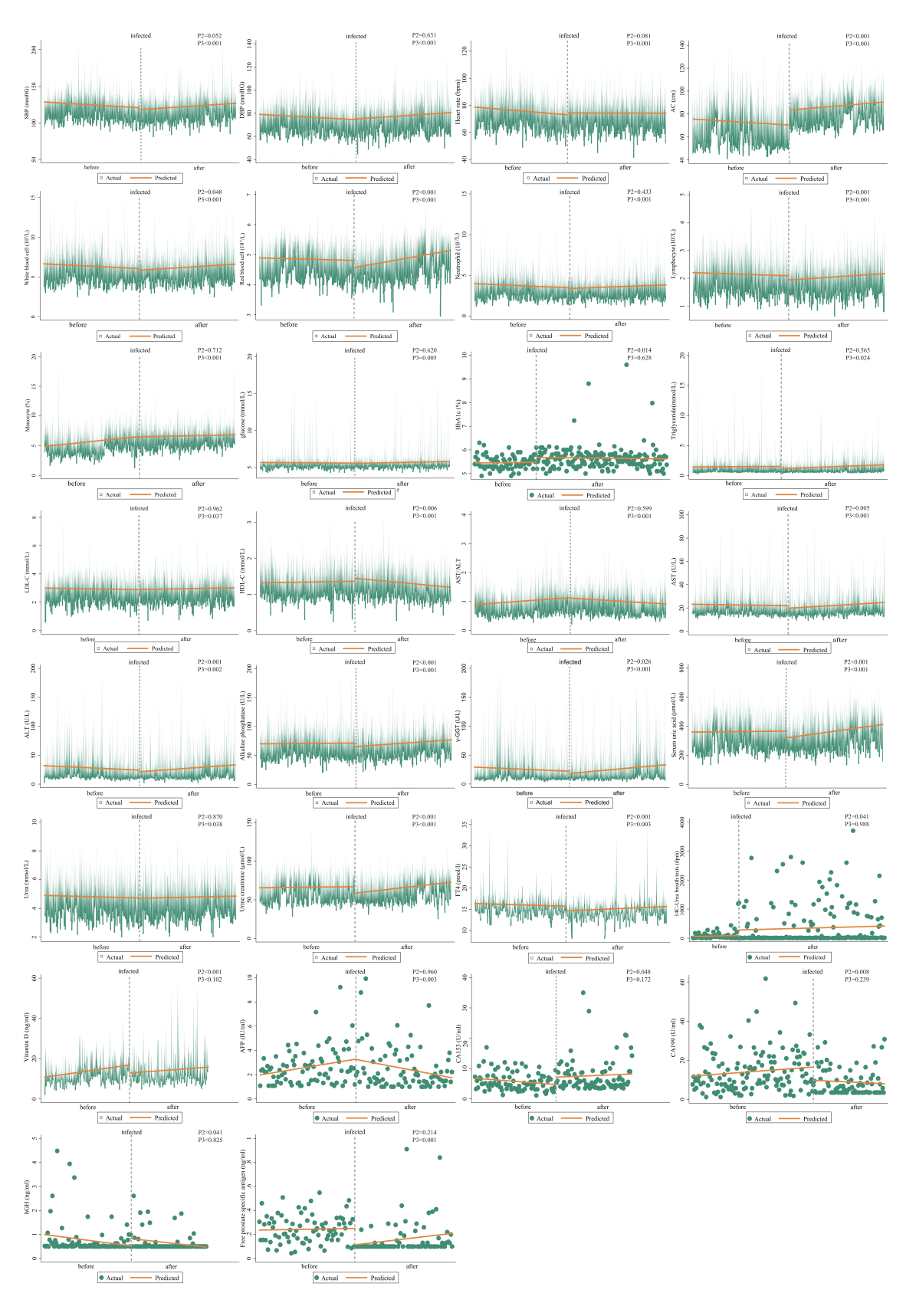


Fig S6 Trends in biomarker levels before and after acute infection in the age groups of 20-44

P2 and P3 in the Fig represent the corresponding P values of β_2_ and β_3_ in interrupted time series analysis, respectively. β_2_ and β_3_ estimate the immediate change and trend in the level of biomarkers after infection, respectively. Green lines and dots represent the actual values of biomarkers, gray dotted lines represent infection, and orange solid lines represent fitting lines for the average value of biomarkers.

*Abbreviations*: *SBP* systolic blood pressure, *DBP* diastolic blood pressure, *AC* abdominal circumference, *HbA1c* glycosylated hemoglobin, *LDL-C* low density lipoprotein cholesterol, *HDL-C* high density lipoprotein cholesterol, *AST/ALT* aspartate aminotransferase/alanine aminotransferase, *AST* aspartate aminotransferase, *ALT* alanine aminotransferase, *γ-GGT* gamma-glutamyl transpeptidase, *FT4* free thyroxine, *AFP* alphafetoprotein, *CA153* cancer antigen 153, *CA199* cancer antigen 199, *hGH* Human growth hormone.

**Fig S7** revealed that 7 biomarkers in the age groups of 45-64 had a significant immediate change after infection (AC, HbA1c, lactate dehydrogenase, urine creatinine, 14C-Urea breath tests, ESR, CA199, P<0.05), and 23 biomarkers had a long-term abnormal trend significantly (SBP, DBP, heart rate, AC, white blood cell, red blood cell, neutrophil %, neutrophil, lymphocyte, monocyte%, monocyte, glucose, triglyceride, HDL-C, AST/ALT, AST, ALT, alkaline phosphatase, γ-GGT, serum uric acid, urea, urine creatinine, CEA, P<0.05).


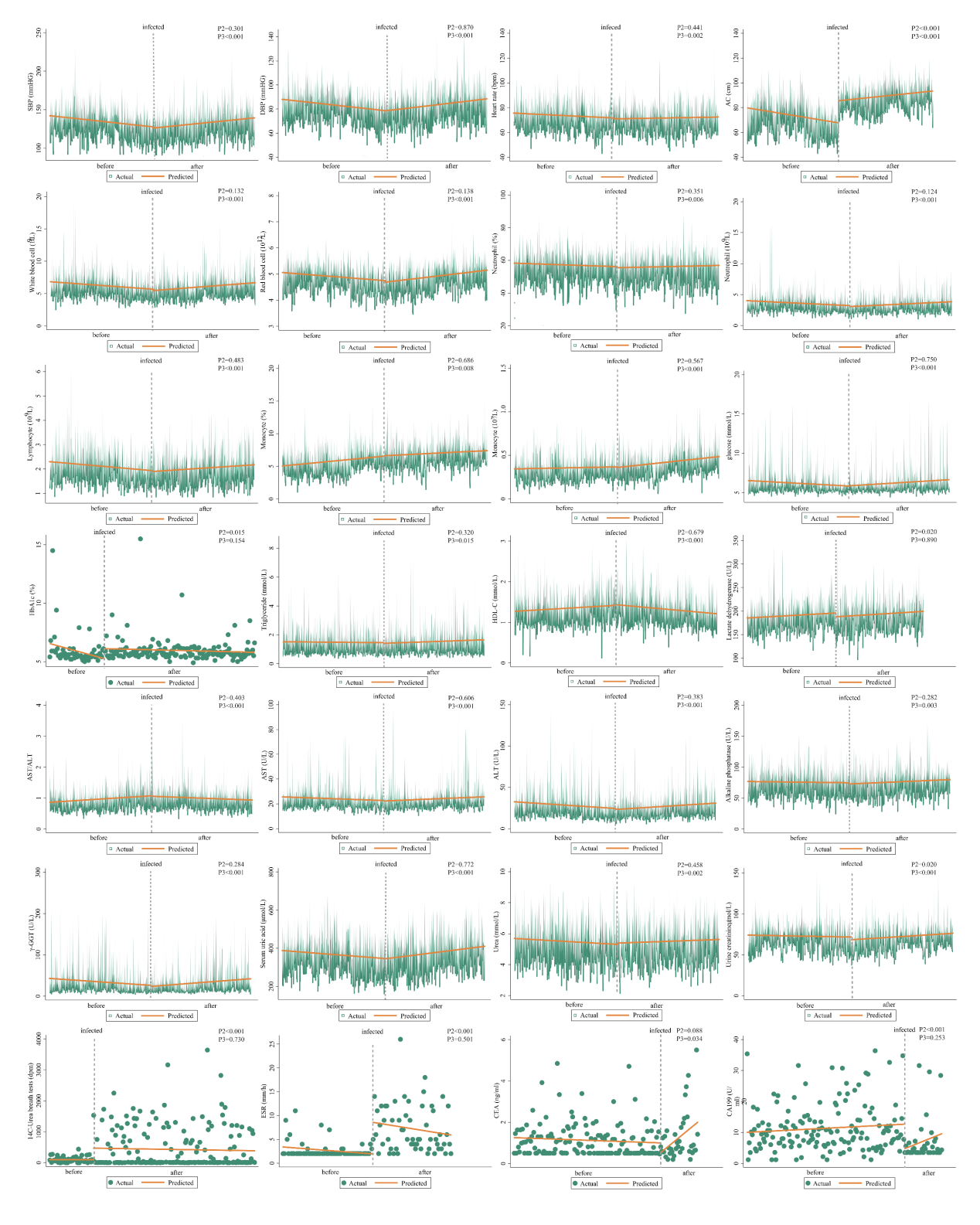


Fig S7 Trends in biomarker levels before and after acute infection in the age groups of 45-64

P2 and P3 in the Fig represent the corresponding P values of β_2_ and β_3_ in interrupted time series analysis, respectively. β_2_ and β_3_ estimate the immediate change and trend in the level of biomarkers after infection, respectively. Green lines and dots represent the actual values of biomarkers, gray dotted lines represent infection, and orange solid lines represent fitting lines for the average value of biomarkers.

*Abbreviations*: *SBP* systolic blood pressure, *DBP* diastolic blood pressure, *AC* abdominal circumference, *HbA1c* glycosylated hemoglobin, *HDL-C* high density lipoprotein cholesterol, *AST/ALT* aspartate aminotransferase/alanine aminotransferase, *AST* aspartate aminotransferase, *ALT* alanine aminotransferase, *γ-GGT* gamma-glutamyl transpeptidase, *ESR* erythrocyte sedimentation rate, *CEA* carcinoembryonic antigen, *CA199* cancer antigen 199.

**Fig S8** revealed that 8 biomarkers in the age groups of 65 and above had a significant immediate change after infection (AC, red blood cell, monocyte%, monocyte, AST/ALT, ALT, alkaline phosphataselactate, urea, P<0.05), and 1 biomarker had a long-term abnormal trend significantly (AST/ALT, P<0.05).


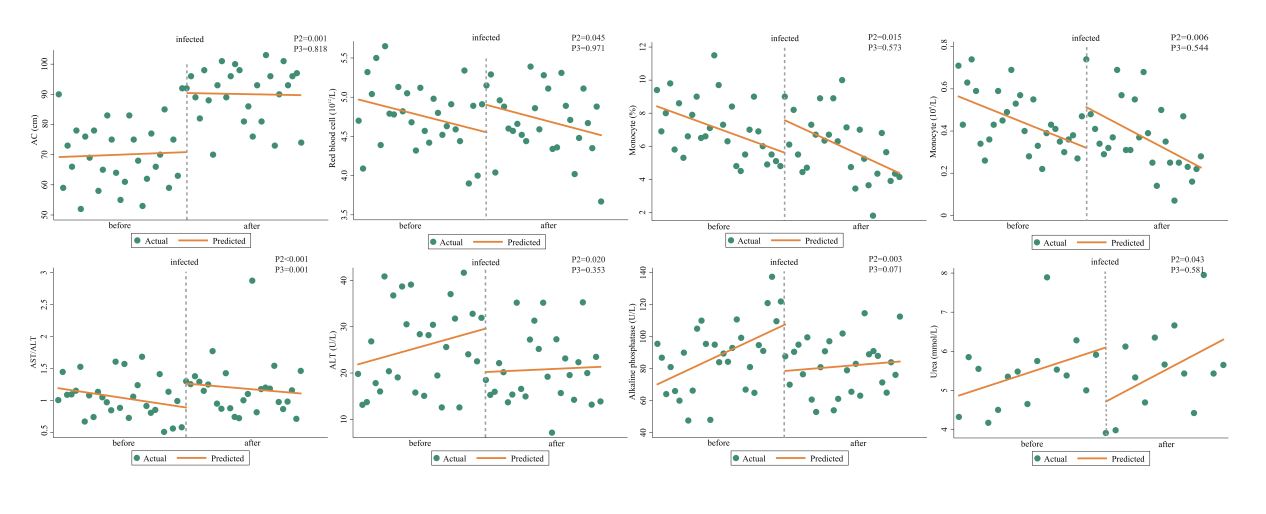


Fig S8 Trends in biomarker levels before and after acute infection in the age groups of 65 and above.

P2 and P3 in the Fig represent the corresponding P values of β_2_ and β_3_ in interrupted time series analysis, respectively. β_2_ and β_3_ estimate the immediate change and trend in the level of biomarkers after infection, respectively. Green lines and dots represent the actual values of biomarkers, gray dotted lines represent infection, and orange solid lines represent fitting lines for the average value of biomarkers.

*Abbreviations*: *AC* abdominal circumference, *AST/ALT* aspartate aminotransferase/alanine aminotransferase, *ALT* alanine aminotransferase.

In the age groups of 20-44, 45-64, and 65 and above, there were 15, 18 and 26 biomarkers have no significant statistical significance in the immediate changes before and after infection and the long-term effects after infection, respectively. (**Table S4**)

**Table S4** Model estimates of level and trend change using interrupted time series analyses for the primary outcomes by age

| Variables | β | 20-44 | | | | | |  | 45-64 | | | | | |  | 65 and above | | | | | |
| --- | --- | --- | --- | --- | --- | --- | --- | --- | --- | --- | --- | --- | --- | --- | --- | --- | --- | --- | --- | --- | --- |
|  |  | Coef. | Std. Err. | t | P value | lower 95%CI | upper 95%CI |  | Coef. | Std. Err. | t | P value | lower 95%CI | upper 95%CI |  | Coef. | Std. Err. | t | P value | lower 95%CI | upper 95%CI |
| SBP  (mmHG) | β0 | - | - | - | - | - | - |  | - | - | - | - | - | - |  | 140.03 | 8.36 | 16.75 | <0.001 | 123.26 | 156.81 |
|  | β1 | - | - | - | - | - | - |  | - | - | - | - | - | - |  | -0.06 | 0.46 | -0.13 | 0.89 | -0.98 | 0.85 |
|  | β2 | - | - | - | - | - | - |  | - | - | - | - | - | - |  | 4.56 | 10.43 | 0.44 | 0.66 | -16.37 | 25.50 |
|  | β3 | - | - | - | - | - | - |  | - | - | - | - | - | - |  | -0.01 | 0.72 | -0.02 | 0.99 | -1.45 | 1.43 |
| DBP  (mmHG) | β0 | - | - | - | - | - | - |  | - | - | - | - | - | - |  | 79.36 | 2.87 | 27.64 | <0.001 | 73.60 | 85.12 |
|  | β1 | - | - | - | - | - | - |  | - | - | - | - | - | - |  | -0.15 | 0.17 | -0.89 | 0.38 | -0.48 | 0.19 |
|  | β2 | - | - | - | - | - | - |  | - | - | - | - | - | - |  | 6.03 | 4.61 | 1.31 | 0.20 | -3.22 | 15.28 |
|  | β3 | - | - | - | - | - | - |  | - | - | - | - | - | - |  | -0.16 | 0.29 | -0.53 | 0.60 | -0.74 | 0.43 |
| BMI  (kg/m^2^) | β0 | 23.54 | 0.40 | 58.21 | <0.001 | 22.74 | 24.33 |  | 24.22 | 0.41 | 59.72 | <0.001 | 23.43 | 25.02 |  | 26.16 | 0.96 | 27.20 | <0.001 | 24.20 | 28.11 |
|  | β1 | 0.00 | 0.00 | 0.19 | 0.85 | 0.00 | 0.00 |  | 0.00 | 0.00 | -0.82 | 0.41 | -0.01 | 0.00 |  | -0.09 | 0.14 | -0.66 | 0.51 | -0.37 | 0.19 |
|  | β2 | -0.04 | 0.48 | -0.09 | 0.93 | -0.99 | 0.90 |  | 0.70 | 0.39 | 1.81 | 0.07 | -0.06 | 1.47 |  | -0.18 | 1.47 | -0.12 | 0.90 | -3.17 | 2.81 |
|  | β3 | 0.00 | 0.00 | 1.13 | 0.26 | 0.00 | 0.01 |  | 0.00 | 0.00 | 1.63 | 0.10 | 0.00 | 0.01 |  | 0.16 | 0.16 | 1.01 | 0.32 | -0.16 | 0.47 |
| Heart rate  (bpm) | β0 | - | - | - | - | - | - |  | - | - | - | - | - | - |  | 78.09 | 4.48 | 17.44 | <0.001 | 69.10 | 87.09 |
|  | β1 | - | - | - | - | - | - |  | - | - | - | - | - | - |  | -0.19 | 0.25 | -0.74 | 0.46 | -0.70 | 0.32 |
|  | β2 | - | - | - | - | - | - |  | - | - | - | - | - | - |  | 4.31 | 5.33 | 0.81 | 0.42 | -6.39 | 15.00 |
|  | β3 | - | - | - | - | - | - |  | - | - | - | - | - | - |  | 0.14 | 0.36 | 0.40 | 0.69 | -0.57 | 0.86 |
| White blood cell  (10^9^/L) | β0 | - | - | - | - | - | - |  | 6.64 | 0.63 | 10.60 | <0.001 | 5.38 | 7.90 |  | 6.64 | 0.63 | 10.60 | <0.001 | 5.38 | 7.90 |
|  | β1 | - | - | - | - | - | - |  | -0.03 | 0.04 | -0.63 | 0.53 | -0.11 | 0.06 |  | -0.03 | 0.04 | -0.63 | 0.53 | -0.11 | 0.06 |
|  | β2 | - | - | - | - | - | - |  | 1.00 | 0.87 | 1.15 | 0.26 | -0.74 | 2.75 |  | 1.00 | 0.87 | 1.15 | 0.26 | -0.74 | 2.75 |
|  | β3 | - | - | - | - | - | - |  | -0.04 | 0.05 | -0.75 | 0.46 | -0.14 | 0.07 |  | -0.04 | 0.05 | -0.75 | 0.46 | -0.14 | 0.07 |
| Neutrophil  (%) | β0 | 58.61 | 0.49 | 119.34 | <0.001 | 57.65 | 59.57 |  | - | - | - | - | - | - |  | 60.18 | 2.78 | 21.67 | <0.001 | 54.61 | 65.75 |
|  | β1 | 0.00 | 0.00 | -2.72 | 0.01 | 0.00 | 0.00 |  | - | - | - | - | - | - |  | -0.03 | 0.16 | -0.17 | 0.86 | -0.35 | 0.29 |
|  | β2 | 0.84 | 0.65 | 1.30 | 0.19 | -0.43 | 2.11 |  | - | - | - | - | - | - |  | 4.68 | 4.04 | 1.16 | 0.25 | -3.42 | 12.78 |
|  | β3 | 0.00 | 0.00 | 1.74 | 0.08 | 0.00 | 0.00 |  | - | - | - | - | - | - |  | -0.35 | 0.25 | -1.37 | 0.18 | -0.86 | 0.16 |
| Neutrophil  (10^9^/L) | β0 | - | - | - | - | - | - |  | - | - | - | - | - | - |  | 4.09 | 0.49 | 8.35 | <0.001 | 3.10 | 5.07 |
|  | β1 | - | - | - | - | - | - |  | - | - | - | - | - | - |  | -0.02 | 0.03 | -0.51 | 0.61 | -0.08 | 0.05 |
|  | β2 | - | - | - | - | - | - |  | - | - | - | - | - | - |  | 0.79 | 0.65 | 1.22 | 0.23 | -0.51 | 2.09 |
|  | β3 | - | - | - | - | - | - |  | - | - | - | - | - | - |  | -0.05 | 0.04 | -1.19 | 0.24 | -0.12 | 0.03 |
| Lymphocyte  (%) | β0 | 34.20 | 0.46 | 74.78 | <0.001 | 33.30 | 35.09 |  | 34.51 | 0.48 | 72.36 | <0.001 | 33.58 | 35.45 |  | 29.70 | 2.74 | 10.84 | <0.001 | 24.21 | 35.20 |
|  | β1 | 0.00 | 0.00 | 0.86 | 0.39 | 0.00 | 0.00 |  | 0.00 | 0.00 | 0.12 | 0.90 | 0.00 | 0.00 |  | 0.03 | 0.17 | 0.19 | 0.85 | -0.31 | 0.37 |
|  | β2 | -1.04 | 0.60 | -1.73 | 0.08 | -2.23 | 0.14 |  | 0.46 | 0.66 | 0.71 | 0.48 | -0.82 | 1.75 |  | -4.37 | 3.98 | -1.10 | 0.28 | -12.36 | 3.62 |
|  | β3 | 0.00 | 0.00 | -0.88 | 0.38 | 0.00 | 0.00 |  | 0.00 | 0.00 | -1.76 | 0.08 | 0.00 | 0.00 |  | 0.42 | 0.24 | 1.74 | 0.09 | -0.06 | 0.90 |
| Lymphocyte  (10^9^/L) | β0 | - | - | - | - | - | - |  | - | - | - | - | - | - |  | 1.96 | 0.19 | 10.49 | <0.001 | 1.59 | 2.34 |
|  | β1 | - | - | - | - | - | - |  | - | - | - | - | - | - |  | 0.00 | 0.01 | -0.49 | 0.62 | -0.02 | 0.02 |
|  | β2 | - | - | - | - | - | - |  | - | - | - | - | - | - |  | 0.01 | 0.30 | 0.03 | 0.98 | -0.59 | 0.61 |
|  | β3 | - | - | - | - | - | - |  | - | - | - | - | - | - |  | 0.01 | 0.02 | 0.66 | 0.52 | -0.02 | 0.05 |
| Monocyte  (10^9^/L) | β0 | 0.32 | 0.01 | 42.80 | <0.001 | 0.30 | 0.33 |  | - | - | - | - | - | - |  | - | - | - | - | - | - |
|  | β1 | 0.00 | 0.00 | 5.70 | <0.001 | 0.00 | 0.00 |  | - | - | - | - | - | - |  | - | - | - | - | - | - |
|  | β2 | -0.02 | 0.01 | -1.63 | 0.10 | -0.04 | 0.00 |  | - | - | - | - | - | - |  | - | - | - | - | - | - |
|  | β3 | 0.00 | 0.00 | -0.01 | 0.99 | 0.00 | 0.00 |  | - | - | - | - | - | - |  | - | - | - | - | - | - |
| FBG  (mmol/L) | β0 | - | - | - | - | - | - |  | - | - | - | - | - | - |  | 6.45 | 0.50 | 12.92 | <0.001 | 5.45 | 7.45 |
|  | β1 | - | - | - | - | - | - |  | - | - | - | - | - | - |  | 0.04 | 0.04 | 0.82 | 0.42 | -0.05 | 0.12 |
|  | β2 | - | - | - | - | - | - |  | - | - | - | - | - | - |  | -0.59 | 1.14 | -0.52 | 0.61 | -2.87 | 1.69 |
|  | β3 | - | - | - | - | - | - |  | - | - | - | - | - | - |  | -0.06 | 0.06 | -1.00 | 0.32 | -0.17 | 0.06 |
| Triglyceride  (mmol/L) | β0 | - | - | - | - | - | - |  | - | - | - | - | - | - |  | 1.45 | 0.24 | 6.08 | <0.001 | 0.97 | 1.92 |
|  | β1 | - | - | - | - | - | - |  | - | - | - | - | - | - |  | 0.03 | 0.01 | 2.70 | 0.01 | 0.01 | 0.05 |
|  | β2 | - | - | - | - | - | - |  | - | - | - | - | - | - |  | -0.27 | 0.62 | -0.44 | 0.66 | -1.52 | 0.97 |
|  | β3 | - | - | - | - | - | - |  | - | - | - | - | - | - |  | -0.05 | 0.03 | -1.58 | 0.12 | -0.11 | 0.01 |
| Total cholesterol  (mmol/L) | β0 | 5.06 | 0.05 | 97.82 | <0.001 | 4.96 | 5.16 |  | 5.40 | 0.06 | 88.13 | <0.001 | 5.28 | 5.52 |  | 4.94 | 0.39 | 12.74 | <0.001 | 4.16 | 5.72 |
|  | β1 | 0.00 | 0.00 | -0.67 | 0.50 | 0.00 | 0.00 |  | 0.00 | 0.00 | 0.24 | 0.81 | 0.00 | 0.00 |  | 0.01 | 0.02 | 0.65 | 0.52 | -0.03 | 0.06 |
|  | β2 | -0.02 | 0.07 | -0.23 | 0.82 | -0.16 | 0.13 |  | 0.10 | 0.09 | 1.05 | 0.29 | -0.08 | 0.28 |  | -0.06 | 0.49 | -0.11 | 0.91 | -1.05 | 0.94 |
|  | β3 | 0.00 | 0.00 | 1.39 | 0.16 | 0.00 | 0.00 |  | 0.00 | 0.00 | -1.76 | 0.08 | 0.00 | 0.00 |  | -0.05 | 0.03 | -1.95 | 0.06 | -0.11 | 0.00 |
| LDL-C  (mmol/L) | β0 | - | - | - | - | - | - |  | 3.30 | 0.05 | 63.07 | <0.001 | 3.20 | 3.40 |  | 2.70 | 0.32 | 8.35 | <0.001 | 2.05 | 3.34 |
|  | β1 | - | - | - | - | - | - |  | 0.00 | 0.00 | -0.45 | 0.66 | 0.00 | 0.00 |  | 0.02 | 0.02 | 1.19 | 0.24 | -0.02 | 0.06 |
|  | β2 | - | - | - | - | - | - |  | 0.05 | 0.08 | 0.66 | 0.51 | -0.10 | 0.20 |  | -0.51 | 0.50 | -1.02 | 0.31 | -1.51 | 0.50 |
|  | β3 | - | - | - | - | - | - |  | 0.00 | 0.00 | -1.03 | 0.30 | 0.00 | 0.00 |  | -0.04 | 0.03 | -1.37 | 0.18 | -0.09 | 0.02 |
| HDL-C  (mmol/L) | β0 | - | - | - | - | - | - |  | - | - | - | - | - | - |  | 1.53 | 0.12 | 12.40 | <0.001 | 1.28 | 1.78 |
|  | β1 | - | - | - | - | - | - |  | - | - | - | - | - | - |  | -0.01 | 0.01 | -2.30 | 0.03 | -0.03 | 0.00 |
|  | β2 | - | - | - | - | - | - |  | - | - | - | - | - | - |  | 0.28 | 0.18 | 1.55 | 0.13 | -0.08 | 0.65 |
|  | β3 | - | - | - | - | - | - |  | - | - | - | - | - | - |  | 0.01 | 0.01 | 0.90 | 0.37 | -0.01 | 0.03 |
| Total bilirubin  (μmol/L) | β0 | 13.69 | 0.32 | 42.38 | <0.001 | 13.06 | 14.33 |  | 14.09 | 0.32 | 43.61 | <0.001 | 13.46 | 14.73 |  | - | - | - | - | - | - |
|  | β1 | 0.00 | 0.00 | -0.06 | 0.95 | 0.00 | 0.00 |  | 0.00 | 0.00 | 0.24 | 0.81 | 0.00 | 0.00 |  | - | - | - | - | - | - |
|  | β2 | -0.24 | 0.45 | -0.53 | 0.60 | -1.13 | 0.65 |  | -0.78 | 0.45 | -1.72 | 0.09 | -1.67 | 0.11 |  | - | - | - | - | - | - |
|  | β3 | 0.00 | 0.00 | -0.36 | 0.72 | 0.00 | 0.00 |  | 0.00 | 0.00 | 1.12 | 0.26 | 0.00 | 0.00 |  | - | - | - | - | - | - |
| Lactate dehydrogenase (U/L) | β0 | 182.53 | 2.45 | 74.61 | <0.001 | 177.73 | 187.33 |  | - | - | - | - | - | - |  | - | - | - | - | - | - |
|  | β1 | 0.01 | 0.01 | 1.05 | 0.30 | -0.01 | 0.02 |  | - | - | - | - | - | - |  | - | - | - | - | - | - |
|  | β2 | -5.38 | 3.69 | -1.46 | 0.15 | -12.61 | 1.85 |  | - | - | - | - | - | - |  | - | - | - | - | - | - |
|  | β3 | 0.01 | 0.01 | 0.57 | 0.57 | -0.01 | 0.03 |  | - | - | - | - | - | - |  | - | - | - | - | - | - |
| AST  (U/L) | β0 | - | - | - | - | - | - |  | - | - | - | - | - | - |  | 26.83 | 8.69 | 3.09 | 0.00 | 9.38 | 44.27 |
|  | β1 | - | - | - | - | - | - |  | - | - | - | - | - | - |  | 0.20 | 0.43 | 0.48 | 0.64 | -0.65 | 1.06 |
|  | β2 | - | - | - | - | - | - |  | - | - | - | - | - | - |  | 1.57 | 12.25 | 0.13 | 0.90 | -23.01 | 26.15 |
|  | β3 | - | - | - | - | - | - |  | - | - | - | - | - | - |  | -0.79 | 0.68 | -1.15 | 0.25 | -2.16 | 0.58 |
| γ-GGT  (U/L) | β0 | - | - | - | - | - | - |  | - | - | - | - | - | - |  | 26.83 | 8.69 | 3.09 | 0.00 | 9.38 | 44.27 |
|  | β1 | - | - | - | - | - | - |  | - | - | - | - | - | - |  | 0.20 | 0.43 | 0.48 | 0.64 | -0.65 | 1.06 |
|  | β2 | - | - | - | - | - | - |  | - | - | - | - | - | - |  | 1.57 | 12.25 | 0.13 | 0.90 | -23.01 | 26.15 |
|  | β3 | - | - | - | - | - | - |  | - | - | - | - | - | - |  | -0.79 | 0.68 | -1.15 | 0.25 | -2.16 | 0.58 |
| Serum uric acid  (μmol/L) | β0 | - | - | - | - | - | - |  | - | - | - | - | - | - |  | 401.09 | 23.06 | 17.39 | <0.001 | 354.81 | 447.37 |
|  | β1 | - | - | - | - | - | - |  | - | - | - | - | - | - |  | -1.94 | 1.25 | -1.54 | 0.13 | -4.45 | 0.58 |
|  | β2 | - | - | - | - | - | - |  | - | - | - | - | - | - |  | 49.48 | 30.59 | 1.62 | 0.11 | -11.90 | 110.86 |
|  | β3 | - | - | - | - | - | - |  | - | - | - | - | - | - |  | 0.58 | 1.86 | 0.31 | 0.76 | -3.14 | 4.31 |
| Urine creatinine  (μmol/L) | β0 | - | - | - | - | - | - |  | - | - | - | - | - | - |  | 72.42 | 7.06 | 10.25 | <0.001 | 57.84 | 86.99 |
|  | β1 | - | - | - | - | - | - |  | - | - | - | - | - | - |  | 0.49 | 0.75 | 0.65 | 0.52 | -1.07 | 2.04 |
|  | β2 | - | - | - | - | - | - |  | - | - | - | - | - | - |  | -6.46 | 9.86 | -0.65 | 0.52 | -26.81 | 13.90 |
|  | β3 | - | - | - | - | - | - |  | - | - | - | - | - | - |  | -0.26 | 1.17 | -0.23 | 0.82 | -2.67 | 2.15 |
| FT4  (pmol/l) | β0 | - | - | - | - | - | - |  | 15.81 | 0.41 | 38.89 | <0.001 | 15.01 | 16.61 |  | - | - | - | - | - | - |
|  | β1 | - | - | - | - | - | - |  | 0.00 | 0.01 | -0.32 | 0.75 | -0.01 | 0.01 |  | - | - | - | - | - | - |
|  | β2 | - | - | - | - | - | - |  | -1.04 | 0.70 | -1.48 | 0.14 | -2.42 | 0.35 |  | - | - | - | - | - | - |
|  | β3 | - | - | - | - | - | - |  | 0.01 | 0.01 | 1.08 | 0.28 | -0.01 | 0.03 |  | - | - | - | - | - | - |
| sTSH  (mIU/L) | β0 | 2.25 | 0.13 | 17.83 | <0.001 | 2.00 | 2.50 |  | 3.33 | 1.99 | 1.67 | 0.10 | -0.59 | 7.25 |  | - | - | - | - | - | - |
|  | β1 | 0.00 | 0.00 | -1.17 | 0.24 | 0.00 | 0.00 |  | 0.00 | 0.02 | 0.09 | 0.93 | -0.04 | 0.05 |  | - | - | - | - | - | - |
|  | β2 | 0.34 | 0.35 | 0.98 | 0.33 | -0.35 | 1.03 |  | -2.20 | 1.79 | -1.23 | 0.22 | -5.73 | 1.33 |  | - | - | - | - | - | - |
|  | β3 | 0.00 | 0.00 | 0.15 | 0.88 | 0.00 | 0.00 |  | 0.02 | 0.03 | 0.60 | 0.55 | -0.04 | 0.08 |  | - | - | - | - | - | - |
| anti tg  (U/ml) | β0 | 229.06 | 39.01 | 5.87 | <0.001 | 152.49 | 305.64 |  | 138.02 | 58.94 | 2.34 | 0.02 | 21.95 | 254.09 |  | - | - | - | - | - | - |
|  | β1 | -0.40 | 0.15 | -2.66 | 0.01 | -0.70 | -0.11 |  | 0.33 | 0.91 | 0.37 | 0.72 | -1.46 | 2.13 |  | - | - | - | - | - | - |
|  | β2 | 81.20 | 42.35 | 1.92 | 0.06 | -1.93 | 164.32 |  | -38.92 | 86.94 | -0.45 | 0.66 | -210.14 | 132.29 |  | - | - | - | - | - | - |
|  | β3 | 0.38 | 0.20 | 1.89 | 0.06 | -0.01 | 0.77 |  | 0.03 | 1.16 | 0.03 | 0.98 | -2.26 | 2.32 |  | - | - | - | - | - | - |
| anti tpo  (U/ml) | β0 | 54.58 | 7.52 | 7.26 | <0.001 | 39.83 | 69.33 |  | 42.10 | 11.69 | 3.60 | <0.001 | 19.07 | 65.13 |  | - | - | - | - | - | - |
|  | β1 | -0.07 | 0.03 | -2.27 | 0.02 | -0.13 | -0.01 |  | 0.04 | 0.15 | 0.27 | 0.79 | -0.26 | 0.35 |  | - | - | - | - | - | - |
|  | β2 | 18.42 | 9.67 | 1.91 | 0.06 | -0.55 | 37.40 |  | -4.70 | 15.33 | -0.31 | 0.76 | -34.90 | 25.49 |  | - | - | - | - | - | - |
|  | β3 | 0.01 | 0.04 | 0.29 | 0.78 | -0.07 | 0.09 |  | -0.22 | 0.19 | -1.18 | 0.24 | -0.59 | 0.15 |  | - | - | - | - | - | - |
| ESR  (mm/h) | β0 | 2.62 | 0.77 | 3.39 | 0.00 | 1.06 | 4.17 |  | - | - | - | - | - | - |  | - | - | - | - | - | - |
|  | β1 | 0.06 | 0.06 | 0.97 | 0.34 | -0.06 | 0.17 |  | - | - | - | - | - | - |  | - | - | - | - | - | - |
|  | β2 | 1.19 | 2.20 | 0.54 | 0.59 | -3.24 | 5.62 |  | - | - | - | - | - | - |  | - | - | - | - | - | - |
|  | β3 | 0.04 | 0.19 | 0.18 | 0.86 | -0.36 | 0.43 |  | - | - | - | - | - | - |  | - | - | - | - | - | - |
| Vitamin D  (ng/ml) | β0 | - | - | - | - | - | - |  | 13.85 | 0.97 | 14.33 | <0.001 | 11.95 | 15.75 |  | - | - | - | - | - | - |
|  | β1 | - | - | - | - | - | - |  | 0.03 | 0.01 | 3.37 | 0.00 | 0.01 | 0.04 |  | - | - | - | - | - | - |
|  | β2 | - | - | - | - | - | - |  | -1.08 | 2.67 | -0.40 | 0.69 | -6.33 | 4.18 |  | - | - | - | - | - | - |
|  | β3 | - | - | - | - | - | - |  | -0.02 | 0.05 | -0.35 | 0.73 | -0.11 | 0.08 |  | - | - | - | - | - | - |
| AFP  (IU/ml) | β0 | - | - | - | - | - | - |  | 2.55 | 0.34 | 7.57 | <0.001 | 1.88 | 3.21 |  | - | - | - | - | - | - |
|  | β1 | - | - | - | - | - | - |  | 0.01 | 0.00 | 1.62 | 0.11 | 0.00 | 0.01 |  | - | - | - | - | - | - |
|  | β2 | - | - | - | - | - | - |  | -0.69 | 0.75 | -0.92 | 0.36 | -2.17 | 0.79 |  | - | - | - | - | - | - |
|  | β3 | - | - | - | - | - | - |  | 0.01 | 0.03 | 0.32 | 0.75 | -0.05 | 0.07 |  | - | - | - | - | - | - |
| CEA  (ng/ml) | β0 | 1.11 | 0.13 | 8.57 | <0.001 | 0.85 | 1.36 |  | - | - | - | - | - | - |  | - | - | - | - | - | - |
|  | β1 | 0.00 | 0.00 | -1.02 | 0.31 | -0.01 | 0.00 |  | - | - | - | - | - | - |  | - | - | - | - | - | - |
|  | β2 | 0.08 | 0.19 | 0.40 | 0.69 | -0.30 | 0.46 |  | - | - | - | - | - | - |  | - | - | - | - | - | - |
|  | β3 | 0.01 | 0.00 | 1.43 | 0.15 | 0.00 | 0.01 |  | - | - | - | - | - | - |  | - | - | - | - | - | - |
| CA153  (U/ml) | β0 | - | - | - | - | - | - |  | 7.21 | 0.56 | 12.97 | <0.001 | 6.11 | 8.31 |  | - | - | - | - | - | - |
|  | β1 | - | - | - | - | - | - |  | -0.01 | 0.01 | -1.59 | 0.11 | -0.03 | 0.00 |  | - | - | - | - | - | - |
|  | β2 | - | - | - | - | - | - |  | 6.03 | 3.27 | 1.84 | 0.07 | -0.43 | 12.49 |  | - | - | - | - | - | - |
|  | β3 | - | - | - | - | - | - |  | -0.14 | 0.14 | -1.04 | 0.30 | -0.42 | 0.13 |  | - | - | - | - | - | - |
| CA242  (U/ml) | β0 | 1.95 | 0.46 | 4.25 | <0.001 | 1.04 | 2.85 |  | 1.86 | 0.37 | 5.04 | <0.001 | 1.13 | 2.59 |  | - | - | - | - | - | - |
|  | β1 | 0.02 | 0.01 | 2.17 | 0.03 | 0.00 | 0.04 |  | 0.02 | 0.01 | 1.29 | 0.20 | -0.01 | 0.04 |  | - | - | - | - | - | - |
|  | β2 | -0.87 | 0.67 | -1.30 | 0.20 | -2.19 | 0.45 |  | -0.69 | 0.62 | -1.12 | 0.27 | -1.92 | 0.54 |  | - | - | - | - | - | - |
|  | β3 | -0.01 | 0.01 | -0.89 | 0.37 | -0.04 | 0.01 |  | 0.00 | 0.00 | 0.14 | 0.89 | 0.00 | 0.00 |  | - | - | - | - | - | - |
| Serum iron  (ng/ml) | β0 | 101.75 | 23.90 | 4.26 | <0.001 | 54.57 | 148.93 |  | 139.46 | 28.13 | 4.96 | <0.001 | 83.56 | 195.36 |  | - | - | - | - | - | - |
|  | β1 | 0.07 | 0.47 | 0.14 | 0.89 | -0.86 | 0.99 |  | -1.60 | 0.93 | -1.72 | 0.09 | -3.46 | 0.25 |  | - | - | - | - | - | - |
|  | β2 | -5.85 | 34.94 | -0.17 | 0.87 | -74.84 | 63.14 |  | 58.44 | 44.41 | 1.32 | 0.19 | -29.82 | 146.70 |  | - | - | - | - | - | - |
|  | β3 | 0.29 | 0.72 | 0.40 | 0.69 | -1.13 | 1.70 |  | 0.52 | 1.80 | 0.29 | 0.78 | -3.06 | 4.09 |  | - | - | - | - | - | - |
| βHCG  (mIU/m) | β0 | 0.34 | 0.01 | 23.54 | <0.001 | 0.31 | 0.37 |  | 0.34 | 0.02 | 16.83 | <0.001 | 0.30 | 0.38 |  | - | - | - | - | - | - |
|  | β1 | 0.00 | 0.00 | 0.20 | 0.84 | 0.00 | 0.00 |  | 0.00 | 0.00 | 0.03 | 0.98 | 0.00 | 0.00 |  | - | - | - | - | - | - |
|  | β2 | -0.02 | 0.02 | -0.70 | 0.48 | -0.06 | 0.03 |  | -0.06 | 0.03 | -1.88 | 0.06 | -0.12 | 0.00 |  | - | - | - | - | - | - |
|  | β3 | 0.00 | 0.00 | -0.57 | 0.57 | 0.00 | 0.00 |  | 0.00 | 0.00 | 1.77 | 0.08 | 0.00 | 0.00 |  | - | - | - | - | - | - |
| hGH  (ng/ml) | β0 | - | - | - | - | - | - |  | 0.56 | 0.14 | 4.15 | <0.001 | 0.29 | 0.83 |  | - | - | - | - | - | - |
|  | β1 | - | - | - | - | - | - |  | 0.00 | 0.00 | 1.10 | 0.28 | 0.00 | 0.01 |  | - | - | - | - | - | - |
|  | β2 | - | - | - | - | - | - |  | -0.18 | 0.21 | -0.85 | 0.40 | -0.61 | 0.24 |  | - | - | - | - | - | - |
|  | β3 | - | - | - | - | - | - |  | 0.00 | 0.01 | -0.26 | 0.80 | -0.02 | 0.01 |  | - | - | - | - | - | - |
| F-PSA  (ng/ml) | β0 | - | - | - | - | - | - |  | 0.27 | 0.02 | 10.99 | <0.001 | 0.22 | 0.31 |  | - | - | - | - | - | - |
|  | β1 | - | - | - | - | - | - |  | 0.00 | 0.00 | 0.32 | 0.75 | 0.00 | 0.00 |  | - | - | - | - | - | - |
|  | β2 | - | - | - | - | - | - |  | -0.07 | 0.07 | -0.98 | 0.33 | -0.20 | 0.07 |  | - | - | - | - | - | - |
|  | β3 | - | - | - | - | - | - |  | 0.00 | 0.00 | -1.19 | 0.24 | -0.01 | 0.00 |  | - | - | - | - | - | - |

Note Table S3: *CI*: confidence interval, β0: constant term, β1: pre-intervention slope, β2: immediate effect, β3: long-term trend.

*Abbreviate*: *SBP* systolic blood pressure, *DBP* diastolic blood pressure, *BMI* body mass index, *FBG* fasting blood glucose, *LDL-C* low-density lipoprotein cholesterol, *HDL-C* high-density lipoprotein cholesterol, *AST* aspartate transaminase, *γ-GGT* γ-glutamyl transpeptidase, *FT4* free thyroxine, *sTSH* sensitive thyroid stimulating hormone, *anti tg* thyroglobulin antigen ,*anti tpo* thyroid peroxidase antibodies ,*ESR* erythrocyte sedimentation rate, *AFP* alphafetoprotein, *CEA* carcinoembryonic antigen, *CA153* cancer antigen 153, *CA242* cancer antigen 242, *βHCG* β-human chorionic gonadotropin, *hGH* Human growth hormone, *F-PSA* free prostate-specific antigen.

There were 31 and 21 biomarkers have significant statistical significance in the immediate changes before and after infection and the long-term effects after infection in infected and uninfected population. (**Table S5**)

**Table S5** Model estimates of level and trend change using interrupted time series analyses for the infected and uninfected people

| Variables | infected | | | | | | |  | uninfected | | | | | |
| --- | --- | --- | --- | --- | --- | --- | --- | --- | --- | --- | --- | --- | --- | --- |
|  |  | Coef. | Std. Err. | t | P value | lower 95%CI | upper 95%CI |  | Coef. | Std. Err. | t | P value | lower 95%CI | upper 95%CI |
| SBP  (mmHG) | β0 | 131.53 | 0.93 | 141.00 | <0.001 | 129.70 | 133.36 |  | 138.34 | 1.29 | 106.97 | <0.001 | 135.80 | 140.88 |
|  | β1 | -0.01 | 0.00 | -5.38 | <0.001 | -0.01 | 0.00 |  | -0.01 | 0.00 | -5.06 | <0.001 | -0.02 | -0.01 |
|  | β2 | -3.25 | 1.21 | -2.70 | 0.01 | -5.62 | -0.89 |  | -0.59 | 1.85 | -0.32 | 0.75 | -4.22 | 3.05 |
|  | β3 | 0.01 | 0.00 | 8.44 | <0.001 | 0.01 | 0.02 |  | 0.02 | 0.00 | 5.83 | <0.001 | 0.02 | 0.03 |
| DBP  (mmHG) | β0 | 81.21 | 0.62 | 130.98 | <0.001 | 79.99 | 82.42 |  | 85.70 | 0.88 | 97.73 | <0.001 | 83.98 | 87.42 |
|  | β1 | 0.00 | 0.00 | -5.44 | <0.001 | -0.01 | 0.00 |  | -0.01 | 0.00 | -5.26 | <0.001 | -0.01 | -0.01 |
|  | β2 | -0.36 | 0.85 | -0.42 | 0.67 | -2.01 | 1.30 |  | 1.22 | 1.23 | 0.99 | 0.32 | -1.19 | 3.63 |
|  | β3 | 0.01 | 0.00 | 6.95 | <0.001 | 0.01 | 0.01 |  | 0.02 | 0.00 | 6.50 | <0.001 | 0.01 | 0.02 |
| Heart rate  (bpm) | β0 | 77.08 | 0.56 | 137.21 | <0.001 | 75.98 | 78.18 |  | - | - | - | - | - | - |
|  | β1 | 0.00 | 0.00 | -5.76 | <0.001 | -0.01 | 0.00 |  | - | - | - | - | - | - |
|  | β2 | 1.51 | 0.77 | 1.96 | 0.05 | 0.00 | 3.01 |  | - | - | - | - | - | - |
|  | β3 | 0.00 | 0.00 | 3.89 | <0.001 | 0.00 | 0.01 |  | - | - | - | - | - | - |
| AC  (cm) | β0 | 75.51 | 0.98 | 77.39 | <0.001 | 73.60 | 77.42 |  | 80.00 | 1.23 | 65.22 | <0.001 | 77.60 | 82.41 |
|  | β1 | -0.01 | 0.00 | -4.74 | <0.001 | -0.01 | 0.00 |  | -0.01 | 0.00 | -3.72 | <0.001 | -0.02 | -0.01 |
|  | β2 | 15.26 | 1.08 | 14.09 | <0.001 | 13.13 | 17.38 |  | 15.71 | 1.45 | 10.85 | <0.001 | 12.87 | 18.55 |
|  | β3 | 0.01 | 0.00 | 7.52 | <0.001 | 0.01 | 0.02 |  | 0.02 | 0.00 | 4.87 | <0.001 | 0.01 | 0.02 |
| White blood cell  (10^9^/L) | β0 | 6.60 | 0.10 | 69.32 | <0.001 | 6.41 | 6.78 |  | 6.87 | 0.12 | 56.48 | <0.001 | 6.63 | 7.11 |
|  | β1 | 0.00 | 0.00 | -4.98 | <0.001 | 0.00 | 0.00 |  | 0.00 | 0.00 | -4.00 | <0.001 | 0.00 | 0.00 |
|  | β2 | -0.21 | 0.11 | -1.85 | 0.06 | -0.43 | 0.01 |  | -0.17 | 0.16 | -1.06 | 0.29 | -0.49 | 0.15 |
|  | β3 | 0.00 | 0.00 | 7.76 | <0.001 | 0.00 | 0.00 |  | 0.00 | 0.00 | 5.56 | <0.001 | 0.00 | 0.00 |
| Red blood cell  (10^12^/L) | β0 | 4.88 | 0.03 | 170.61 | <0.001 | 4.82 | 4.93 |  | 5.09 | 0.03 | 151.46 | <0.001 | 5.03 | 5.16 |
|  | β1 | 0.00 | 0.00 | -2.67 | 0.01 | 0.00 | 0.00 |  | 0.00 | 0.00 | -4.28 | <0.001 | 0.00 | 0.00 |
|  | β2 | -0.21 | 0.04 | -5.74 | <0.001 | -0.28 | -0.14 |  | 0.00 | 0.05 | -0.10 | 0.92 | -0.10 | 0.09 |
|  | β3 | 0.00 | 0.00 | 10.50 | <0.001 | 0.00 | 0.00 |  | 0.00 | 0.00 | 6.30 | <0.001 | 0.00 | 0.00 |
| Neutrophil  (%) | β0 | - | - | - | - | - | - |  | 58.99 | 0.55 | 107.02 | <0.001 | 57.91 | 60.07 |
|  | β1 | - | - | - | - | - | - |  | 0.00 | 0.00 | -2.88 | 0.00 | -0.01 | 0.00 |
|  | β2 | - | - | - | - | - | - |  | 0.18 | 0.79 | 0.23 | 0.82 | -1.36 | 1.73 |
|  | β3 | - | - | - | - | - | - |  | 0.00 | 0.00 | 2.26 | 0.02 | 0.00 | 0.01 |
| Neutrophil  (10^9^/L) | β0 | 3.90 | 0.08 | 49.47 | <0.001 | 3.75 | 4.05 |  | 4.08 | 0.09 | 43.79 | <0.001 | 3.90 | 4.26 |
|  | β1 | 0.00 | 0.00 | -4.62 | <0.001 | 0.00 | 0.00 |  | 0.00 | 0.00 | -4.05 | <0.001 | 0.00 | 0.00 |
|  | β2 | -0.10 | 0.09 | -1.17 | 0.24 | -0.27 | 0.07 |  | -0.09 | 0.12 | -0.71 | 0.48 | -0.33 | 0.15 |
|  | β3 | 0.00 | 0.00 | 6.57 | <0.001 | 0.00 | 0.00 |  | 0.00 | 0.00 | 4.89 | <0.001 | 0.00 | 0.00 |
| Lymphocyte  (10^9^/L) | β0 | 2.22 | 0.03 | 68.78 | <0.001 | 2.15 | 2.28 |  | 2.27 | 0.04 | 56.25 | <0.001 | 2.19 | 2.35 |
|  | β1 | 0.00 | 0.00 | -4.37 | <0.001 | 0.00 | 0.00 |  | 0.00 | 0.00 | -2.56 | 0.01 | 0.00 | 0.00 |
|  | β2 | -0.08 | 0.04 | -1.99 | 0.05 | -0.16 | 0.00 |  | -0.11 | 0.06 | -1.99 | 0.05 | -0.22 | 0.00 |
|  | β3 | 0.00 | 0.00 | 6.15 | <0.001 | 0.00 | 0.00 |  | 0.00 | 0.00 | 4.02 | <0.001 | 0.00 | 0.00 |
| Monocyte  (%) | β0 | 4.86 | 0.10 | 50.85 | <0.001 | 4.67 | 5.05 |  | 5.16 | 0.13 | 38.75 | <0.001 | 4.90 | 5.42 |
|  | β1 | 0.00 | 0.00 | 11.26 | <0.001 | 0.00 | 0.00 |  | 0.00 | 0.00 | 5.00 | <0.001 | 0.00 | 0.00 |
|  | β2 | -0.23 | 0.13 | -1.69 | 0.09 | -0.49 | 0.04 |  | 0.43 | 0.17 | 2.55 | 0.01 | 0.10 | 0.75 |
|  | β3 | 0.00 | 0.00 | -6.35 | <0.001 | 0.00 | 0.00 |  | 0.00 | 0.00 | -1.79 | 0.07 | 0.00 | 0.00 |
| Monocyte  (10^9^/L) | β0 | 0.32 | 0.01 | 45.37 | <0.001 | 0.30 | 0.33 |  | 0.35 | 0.01 | 36.16 | <0.001 | 0.33 | 0.37 |
|  | β1 | 0.00 | 0.00 | 6.15 | <0.001 | 0.00 | 0.00 |  | 0.00 | 0.00 | 1.37 | 0.17 | 0.00 | 0.00 |
|  | β2 | -0.03 | 0.01 | -2.72 | 0.01 | -0.04 | -0.01 |  | 0.02 | 0.01 | 1.49 | 0.14 | -0.01 | 0.04 |
|  | β3 | 0.00 | 0.00 | 0.39 | 0.70 | 0.00 | 0.00 |  | 0.00 | 0.00 | 2.94 | 0.00 | 0.00 | 0.00 |
| FBG  (mmol/L) | β0 | 5.92 | 0.06 | 95.37 | <0.001 | 5.79 | 6.04 |  | - | - | - | - | - | - |
|  | β1 | 0.00 | 0.00 | -3.23 | 0.00 | 0.00 | 0.00 |  | - | - | - | - | - | - |
|  | β2 | -0.09 | 0.06 | -1.39 | 0.16 | -0.22 | 0.04 |  | - | - | - | - | - | - |
|  | β3 | 0.00 | 0.00 | 5.56 | <0.001 | 0.00 | 0.00 |  | - | - | - | - | - | - |
| HbA1c  (%) | β0 | 5.71 | 0.14 | 40.46 | <0.001 | 5.44 | 5.99 |  | - | - | - | - | - | - |
|  | β1 | 0.00 | 0.00 | -0.89 | 0.37 | -0.01 | 0.00 |  | - | - | - | - | - | - |
|  | β2 | 0.33 | 0.13 | 2.66 | 0.01 | 0.09 | 0.58 |  | - | - | - | - | - | - |
|  | β3 | 0.00 | 0.00 | 0.51 | 0.61 | 0.00 | 0.01 |  | - | - | - | - | - | - |
| Triglyceride  (mmol/L) | β0 | 1.43 | 0.07 | 20.81 | <0.001 | 1.29 | 1.56 |  | - | - | - | - | - | - |
|  | β1 | 0.00 | 0.00 | 0.48 | 0.63 | 0.00 | 0.00 |  | - | - | - | - | - | - |
|  | β2 | -0.25 | 0.08 | -3.00 | 0.00 | -0.41 | -0.09 |  | - | - | - | - | - | - |
|  | β3 | 0.00 | 0.00 | 2.11 | 0.04 | 0.00 | 0.00 |  | - | - | - | - | - | - |
| HDL-C  (mmol/L) | β0 | 1.29 | 0.02 | 68.89 | <0.001 | 1.26 | 1.33 |  | 1.31 | 0.03 | 42.74 | <0.001 | 1.25 | 1.37 |
|  | β1 | 0.00 | 0.00 | 3.09 | 0.00 | 0.00 | 0.00 |  | 0.00 | 0.00 | 1.15 | 0.25 | 0.00 | 0.00 |
|  | β2 | 0.08 | 0.03 | 2.68 | 0.01 | 0.02 | 0.13 |  | 0.01 | 0.04 | 0.33 | 0.74 | -0.06 | 0.08 |
|  | β3 | 0.00 | 0.00 | -7.64 | <0.001 | 0.00 | 0.00 |  | 0.00 | 0.00 | -3.57 | <0.001 | 0.00 | 0.00 |
| Total bilirubin  (μmol/L) | β0 | 13.56 | 0.28 | 47.80 | <0.001 | 13.01 | 14.12 |  | - | - | - | - | - | - |
|  | β1 | 0.00 | 0.00 | 1.18 | 0.24 | 0.00 | 0.00 |  | - | - | - | - | - | - |
|  | β2 | -1.03 | 0.42 | -2.47 | 0.01 | -1.86 | -0.21 |  | - | - | - | - | - | - |
|  | β3 | 0.00 | 0.00 | 0.31 | 0.76 | 0.00 | 0.00 |  | - | - | - | - | - | - |
| ALT/AST | β0 | 0.92 | 0.02 | 38.78 | <0.001 | 0.87 | 0.96 |  | 0.86 | 0.02 | 35.04 | <0.001 | 0.81 | 0.90 |
|  | β1 | 0.00 | 0.00 | 5.35 | <0.001 | 0.00 | 0.00 |  | 0.00 | 0.00 | 4.10 | <0.001 | 0.00 | 0.00 |
|  | β2 | 0.01 | 0.04 | 0.32 | 0.75 | -0.06 | 0.08 |  | -0.02 | 0.04 | -0.57 | 0.57 | -0.09 | 0.05 |
|  | β3 | 0.00 | 0.00 | -7.51 | <0.001 | 0.00 | 0.00 |  | 0.00 | 0.00 | -4.39 | <0.001 | 0.00 | 0.00 |
| AST  (U/L) | β0 | 23.80 | 0.53 | 44.57 | <0.001 | 22.75 | 24.85 |  | 26.09 | 0.93 | 28.06 | <0.001 | 24.26 | 27.91 |
|  | β1 | 0.00 | 0.00 | -2.40 | 0.02 | 0.00 | 0.00 |  | 0.00 | 0.00 | -1.75 | 0.08 | -0.01 | 0.00 |
|  | β2 | -1.68 | 0.66 | -2.55 | 0.01 | -2.97 | -0.39 |  | -0.46 | 1.07 | -0.43 | 0.67 | -2.56 | 1.64 |
|  | β3 | 0.00 | 0.00 | 5.50 | <0.001 | 0.00 | 0.01 |  | 0.01 | 0.00 | 2.57 | 0.01 | 0.00 | 0.01 |
| ALT  (U/L) | β0 | 30.86 | 1.14 | 27.13 | <0.001 | 28.63 | 33.09 |  | 35.78 | 1.85 | 19.30 | <0.001 | 32.15 | 39.42 |
|  | β1 | -0.01 | 0.00 | -4.30 | <0.001 | -0.01 | 0.00 |  | -0.01 | 0.00 | -3.22 | 0.00 | -0.02 | 0.00 |
|  | β2 | -2.34 | 1.43 | -1.64 | 0.10 | -5.14 | 0.46 |  | 1.26 | 2.12 | 0.59 | 0.55 | -2.90 | 5.42 |
|  | β3 | 0.01 | 0.00 | 7.06 | <0.001 | 0.01 | 0.02 |  | 0.02 | 0.00 | 3.46 | <0.001 | 0.01 | 0.02 |
| Alkaline phosphatase  (U/L) | β0 | 71.20 | 1.17 | 60.84 | <0.001 | 68.91 | 73.50 |  | - | - | - | - | - | - |
|  | β1 | 0.00 | 0.00 | 0.42 | 0.68 | 0.00 | 0.00 |  | - | - | - | - | - | - |
|  | β2 | -4.83 | 1.57 | -3.07 | 0.00 | -7.91 | -1.75 |  | - | - | - | - | - | - |
|  | β3 | 0.01 | 0.00 | 3.12 | 0.00 | 0.00 | 0.01 |  | - | - | - | - | - | - |
| γ-GGT  (U/L) | β0 | 32.30 | 1.82 | 17.73 | <0.001 | 28.73 | 35.87 |  | 40.95 | 2.73 | 14.99 | <0.001 | 35.59 | 46.30 |
|  | β1 | -0.01 | 0.00 | -3.75 | <0.001 | -0.01 | 0.00 |  | -0.01 | 0.01 | -1.92 | 0.06 | -0.02 | 0.00 |
|  | β2 | -3.60 | 1.87 | -1.93 | 0.05 | -7.26 | 0.07 |  | -5.32 | 3.66 | -1.45 | 0.15 | -12.50 | 1.86 |
|  | β3 | 0.02 | 0.00 | 6.55 | <0.001 | 0.01 | 0.02 |  | 0.03 | 0.01 | 4.06 | <0.001 | 0.02 | 0.05 |
| Serum uric acid  (μmol/L) | β0 | 358.95 | 5.66 | 63.38 | <0.001 | 347.85 | 370.05 |  | 388.57 | 6.48 | 59.94 | <0.001 | 375.85 | 401.28 |
|  | β1 | 0.00 | 0.01 | -0.52 | 0.60 | -0.02 | 0.01 |  | -0.03 | 0.01 | -1.88 | 0.06 | -0.05 | 0.00 |
|  | β2 | -34.06 | 7.07 | -4.82 | <0.001 | -47.92 | -20.20 |  | -5.99 | 9.44 | -0.63 | 0.53 | -24.50 | 12.53 |
|  | β3 | 0.06 | 0.01 | 6.87 | <0.001 | 0.04 | 0.08 |  | 0.08 | 0.02 | 4.40 | <0.001 | 0.05 | 0.12 |
| Urea  (mmol/L) | β0 | 5.14 | 0.07 | 70.67 | <0.001 | 5.00 | 5.28 |  | 5.48 | 0.10 | 57.44 | <0.001 | 5.29 | 5.67 |
|  | β1 | 0.00 | 0.00 | -1.71 | 0.09 | 0.00 | 0.00 |  | 0.00 | 0.00 | -2.23 | 0.03 | 0.00 | 0.00 |
|  | β2 | 0.01 | 0.10 | 0.06 | 0.96 | -0.19 | 0.20 |  | 0.01 | 0.14 | 0.07 | 0.95 | -0.26 | 0.28 |
|  | β3 | 0.00 | 0.00 | 2.25 | 0.03 | 0.00 | 0.00 |  | 0.00 | 0.00 | 2.47 | 0.01 | 0.00 | 0.00 |
| Urine creatinine  (μmol/L) | β0 | 67.67 | 0.96 | 70.72 | <0.001 | 65.79 | 69.55 |  | 72.47 | 1.06 | 68.51 | <0.001 | 70.39 | 74.54 |
|  | β1 | 0.00 | 0.00 | 0.24 | 0.81 | 0.00 | 0.00 |  | 0.00 | 0.00 | -0.47 | 0.64 | -0.01 | 0.00 |
|  | β2 | -6.59 | 1.33 | -4.96 | <0.001 | -9.20 | -3.99 |  | -5.76 | 1.96 | -2.93 | 0.00 | -9.61 | -1.91 |
|  | β3 | 0.01 | 0.00 | 5.00 | <0.001 | 0.01 | 0.01 |  | 0.02 | 0.01 | 2.56 | 0.01 | 0.00 | 0.03 |
| FT4  (pmol/l) | β0 | 16.26 | 0.25 | 64.47 | <0.001 | 15.77 | 16.76 |  | - | - | - | - | - | - |
|  | β1 | 0.00 | 0.00 | -1.83 | 0.07 | 0.00 | 0.00 |  | - | - | - | - | - | - |
|  | β2 | -1.00 | 0.33 | -3.06 | 0.00 | -1.65 | -0.36 |  | - | - | - | - | - | - |
|  | β3 | 0.00 | 0.00 | 2.94 | 0.00 | 0.00 | 0.01 |  | - | - | - | - | - | - |
| anti tpo  (U/ml) | β0 | - | - | - | - | - | - |  | 288.69 | 75.05 | 3.85 | <0.001 | 140.79 | 436.58 |
|  | β1 | - | - | - | - | - | - |  | -2.29 | 0.89 | -2.58 | 0.01 | -4.03 | -0.54 |
|  | β2 | - | - | - | - | - | - |  | 136.17 | 67.32 | 2.02 | 0.04 | 3.52 | 268.82 |
|  | β3 | - | - | - | - | - | - |  | 2.07 | 1.16 | 1.79 | 0.08 | -0.21 | 4.36 |
| 14C-Urea breath tests  (dpm) | β0 | 79.49 | 13.90 | 5.72 | <0.001 | 52.16 | 106.82 |  | - | - | - | - | - | - |
|  | β1 | 0.23 | 0.31 | 0.75 | 0.45 | -0.37 | 0.83 |  | - | - | - | - | - | - |
|  | β2 | 281.36 | 71.62 | 3.93 | <0.001 | 140.54 | 422.17 |  | - | - | - | - | - | - |
|  | β3 | -0.20 | 0.54 | -0.37 | 0.71 | -1.25 | 0.86 |  | - | - | - | - | - | - |
| ESR  (mm/h) | β0 | 2.98 | 0.53 | 5.63 | <0.001 | 1.93 | 4.03 |  | 3.76 | 0.73 | 5.18 | <0.001 | 2.31 | 5.22 |
|  | β1 | 0.00 | 0.01 | -0.35 | 0.73 | -0.03 | 0.02 |  | -0.04 | 0.03 | -1.21 | 0.23 | -0.11 | 0.03 |
|  | β2 | 4.86 | 1.13 | 4.30 | <0.001 | 2.63 | 7.09 |  | 6.28 | 2.36 | 2.66 | 0.01 | 1.56 | 11.00 |
|  | β3 | 0.00 | 0.03 | 0.08 | 0.93 | -0.06 | 0.07 |  | -0.03 | 0.14 | -0.23 | 0.82 | -0.30 | 0.24 |
| Vitamin D  (ng/ml) | β0 | 11.71 | 0.59 | 19.82 | <0.001 | 10.55 | 12.87 |  | 11.40 | 0.92 | 12.43 | <0.001 | 9.59 | 13.21 |
|  | β1 | 0.01 | 0.00 | 5.83 | <0.001 | 0.01 | 0.02 |  | 0.06 | 0.02 | 3.64 | <0.001 | 0.03 | 0.09 |
|  | β2 | -3.57 | 1.09 | -3.29 | 0.00 | -5.70 | -1.44 |  | -6.75 | 2.27 | -2.97 | 0.00 | -11.22 | -2.27 |
|  | β3 | -0.01 | 0.00 | -2.55 | 0.01 | -0.02 | 0.00 |  | 0.05 | 0.06 | 0.82 | 0.41 | -0.06 | 0.15 |
| AFP  (IU/ml) | β0 | 2.14 | 0.19 | 11.48 | <0.001 | 1.77 | 2.51 |  | - | - | - | - | - | - |
|  | β1 | 0.01 | 0.00 | 3.74 | <0.001 | 0.00 | 0.01 |  | - | - | - | - | - | - |
|  | β2 | -0.32 | 0.54 | -0.61 | 0.55 | -1.38 | 0.73 |  | - | - | - | - | - | - |
|  | β3 | -0.02 | 0.01 | -2.73 | 0.01 | -0.04 | -0.01 |  | - | - | - | - | - | - |
| CA153  (U/ml) | β0 | 6.99 | 0.44 | 16.06 | <0.001 | 6.13 | 7.85 |  | - | - | - | - | - | - |
|  | β1 | -0.01 | 0.00 | -2.79 | 0.01 | -0.02 | 0.00 |  | - | - | - | - | - | - |
|  | β2 | 4.58 | 1.70 | 2.69 | 0.01 | 1.23 | 7.94 |  | - | - | - | - | - | - |
|  | β3 | -0.01 | 0.03 | -0.43 | 0.67 | -0.08 | 0.05 |  | - | - | - | - | - | - |
| CA199  (U/ml) | β0 | 11.72 | 1.19 | 9.86 | <0.001 | 9.38 | 14.06 |  | - | - | - | - | - | - |
|  | β1 | 0.01 | 0.01 | 1.07 | 0.29 | -0.01 | 0.03 |  | - | - | - | - | - | - |
|  | β2 | -6.19 | 1.94 | -3.19 | 0.00 | -10.00 | -2.37 |  | - | - | - | - | - | - |
|  | β3 | -0.02 | 0.03 | -0.54 | 0.59 | -0.09 | 0.05 |  | - | - | - | - | - | - |
| F-PSA  (ng/ml) | β0 | 0.25 | 0.02 | 12.16 | <0.001 | 0.21 | 0.29 |  | - | - | - | - | - | - |
|  | β1 | 0.00 | 0.00 | 0.97 | 0.33 | 0.00 | 0.00 |  | - | - | - | - | - | - |
|  | β2 | -0.14 | 0.04 | -3.54 | <0.001 | -0.23 | -0.06 |  | - | - | - | - | - | - |
|  | β3 | 0.00 | 0.00 | 0.48 | 0.63 | 0.00 | 0.00 |  | - | - | - | - | - | - |

Note: *CI:* confdence interval,$\beta_{0}$ estimates the FI at the beginning of the time series. $\beta_{1}$ estimates the preinfection trend of FI. $\beta_{2}$ estimates the immediate change in the level of FI after infection. $\beta_{3}$ estimates the after infection trend of FI.

*Abbreviations*: *SBP* Systolic blood pressure, *DBP* Diastolic blood pressure, *AC* Abdominal circumference, *FBG* Fasting blood glucose, *HbA1c* Hemoglobin A1c, *HDL-C* High-density lipoprotein cholesterol, *ALT/AST* Aspartate aminotransferase/Alanine aminotransferase, *AST* Aspartate aminotransferase, *ALT* Alanine aminotransferase, *γ-GGT* gamma-glutamyl transpeptidase, *FT4* Free thyroxine; *ESR* Erythrocyte Sedimentation Rate, *AFP* alpha fetoprotein, *CA153* Carbohydrate antigen 153, *CA199* Carbohydrate antigen 199, *F-PSA* Free-Prostate Specific Antigen.

In the age groups of infected and uninfected and above, there were 14 and 24 biomarkers have no significant statistical significance in the immediate changes before and after infection and the long-term effects after infection, respectively. (**Table S6**)

**Table S6** Model estimates of level and trend change using interrupted time series analyses for the infected and uninfected

| Variables | infected | | | | | | |  | uninfected | | | | | |
| --- | --- | --- | --- | --- | --- | --- | --- | --- | --- | --- | --- | --- | --- | --- |
|  |  | Coef. | Std. Err. | t | P value | lower 95%CI | upper 95%CI |  | Coef. | Std. Err. | t | P value | lower 95%CI | upper 95%CI |
| BMI  (kg/m^2^) | β0 | 23.75 | 0.38 | 62.25 | <0.001 | 23.00 | 24.50 |  | 23.52 | 0.56 | 41.95 | <0.001 | 22.42 | 24.62 |
|  | β1 | 0.00 | 0.00 | -0.34 | 0.73 | 0.00 | 0.00 |  | 0.01 | 0.01 | 0.96 | 0.34 | -0.01 | 0.02 |
|  | β2 | 0.17 | 0.38 | 0.45 | 0.65 | -0.58 | 0.92 |  | 0.33 | 0.63 | 0.52 | 0.60 | -0.91 | 1.57 |
|  | β3 | 0.00 | 0.00 | 1.68 | 0.09 | 0.00 | 0.01 |  | 0.00 | 0.01 | -0.56 | 0.58 | -0.02 | 0.01 |
| Heart rate  (bpm) | β0 | - | - | - | - | - | - |  | 76.92 | 0.76 | 100.90 | <0.001 | 75.43 | 78.42 |
|  | β1 | - | - | - | - | - | - |  | 0.00 | 0.00 | -2.07 | 0.04 | -0.01 | 0.00 |
|  | β2 | - | - | - | - | - | - |  | -1.39 | 1.01 | -1.38 | 0.17 | -3.37 | 0.58 |
|  | β3 | - | - | - | - | - | - |  | 0.00 | 0.00 | 1.61 | 0.11 | 0.00 | 0.01 |
| Neutrophil  (%) | β0 | 58.08 | 0.45 | 129.06 | <0.001 | 57.20 | 58.97 |  | - | - | - | - | - | - |
|  | β1 | 0.00 | 0.00 | -2.20 | 0.03 | 0.00 | 0.00 |  | - | - | - | - | - | - |
|  | β2 | 0.16 | 0.60 | 0.26 | 0.79 | -1.02 | 1.33 |  | - | - | - | - | - | - |
|  | β3 | 0.00 | 0.00 | 1.90 | 0.06 | 0.00 | 0.00 |  | - | - | - | - | - | - |
| Lymphocyte  (%) | β0 | 34.66 | 0.42 | 82.87 | <0.001 | 33.84 | 35.48 |  | 33.88 | 0.51 | 66.69 | <0.001 | 32.88 | 34.87 |
|  | β1 | 0.00 | 0.00 | -0.14 | 0.89 | 0.00 | 0.00 |  | 0.00 | 0.00 | 0.98 | 0.33 | 0.00 | 0.00 |
|  | β2 | -0.30 | 0.56 | -0.53 | 0.59 | -1.39 | 0.80 |  | -0.51 | 0.74 | -0.68 | 0.50 | -1.97 | 0.96 |
|  | β3 | 0.00 | 0.00 | -0.57 | 0.57 | 0.00 | 0.00 |  | 0.00 | 0.00 | -1.55 | 0.12 | -0.01 | 0.00 |
| FBG  (mmol/L) | β0 | - | - | - | - | - | - |  | 6.42 | 0.13 | 47.77 | <0.001 | 6.15 | 6.68 |
|  | β1 | - | - | - | - | - | - |  | 0.00 | 0.00 | -2.31 | 0.02 | 0.00 | 0.00 |
|  | β2 | - | - | - | - | - | - |  | 0.24 | 0.17 | 1.42 | 0.16 | -0.09 | 0.57 |
|  | β3 | - | - | - | - | - | - |  | 0.00 | 0.00 | 1.95 | 0.05 | 0.00 | 0.00 |
| HbA1c  (%) | β0 | - | - | - | - | - | - |  | 6.27 | 0.77 | 8.18 | <0.001 | 4.76 | 7.79 |
|  | β1 | - | - | - | - | - | - |  | -0.02 | 0.03 | -0.84 | 0.40 | -0.08 | 0.03 |
|  | β2 | - | - | - | - | - | - |  | 1.01 | 0.57 | 1.77 | 0.08 | -0.12 | 2.14 |
|  | β3 | - | - | - | - | - | - |  | 0.01 | 0.03 | 0.54 | 0.59 | -0.04 | 0.07 |
| Triglyceride  (mmol/L) | β0 | - | - | - | - | - | - |  | 1.60 | 0.09 | 18.38 | <0.001 | 1.43 | 1.77 |
|  | β1 | - | - | - | - | - | - |  | 0.00 | 0.00 | -0.01 | 0.99 | 0.00 | 0.00 |
|  | β2 | - | - | - | - | - | - |  | -0.15 | 0.11 | -1.40 | 0.16 | -0.35 | 0.06 |
|  | β3 | - | - | - | - | - | - |  | 0.00 | 0.00 | 1.69 | 0.09 | 0.00 | 0.00 |
| Total cholesterol  (mmol/L) | β0 | 5.14 | 0.05 | 101.20 | <0.001 | 5.04 | 5.24 |  | 5.33 | 0.07 | 76.04 | <0.001 | 5.20 | 5.47 |
|  | β1 | 0.00 | 0.00 | 0.46 | 0.65 | 0.00 | 0.00 |  | 0.00 | 0.00 | -1.34 | 0.18 | 0.00 | 0.00 |
|  | β2 | 0.00 | 0.07 | -0.06 | 0.95 | -0.15 | 0.14 |  | 0.10 | 0.10 | 0.95 | 0.34 | -0.10 | 0.29 |
|  | β3 | 0.00 | 0.00 | -0.58 | 0.57 | 0.00 | 0.00 |  | 0.00 | 0.00 | 0.66 | 0.51 | 0.00 | 0.00 |
| LDL-C  (mmol/L) | β0 | 3.07 | 0.04 | 71.85 | <0.001 | 2.98 | 3.15 |  | 3.25 | 0.06 | 54.43 | <0.001 | 3.14 | 3.37 |
|  | β1 | 0.00 | 0.00 | -0.27 | 0.79 | 0.00 | 0.00 |  | 0.00 | 0.00 | -1.85 | 0.07 | 0.00 | 0.00 |
|  | β2 | -0.01 | 0.06 | -0.16 | 0.87 | -0.13 | 0.11 |  | 0.08 | 0.08 | 1.00 | 0.32 | -0.08 | 0.25 |
|  | β3 | 0.00 | 0.00 | 0.36 | 0.72 | 0.00 | 0.00 |  | 0.00 | 0.00 | 0.94 | 0.35 | 0.00 | 0.00 |
| Total bilirubin  (μmol/L) | β0 | - | - | - | - | - | - |  | 14.50 | 0.40 | 36.55 | <0.001 | 13.72 | 15.28 |
|  | β1 | - | - | - | - | - | - |  | 0.00 | 0.00 | -1.67 | 0.10 | 0.00 | 0.00 |
|  | β2 | - | - | - | - | - | - |  | 0.35 | 0.50 | 0.69 | 0.49 | -0.64 | 1.33 |
|  | β3 | - | - | - | - | - | - |  | 0.00 | 0.00 | 0.84 | 0.40 | 0.00 | 0.00 |
| Lactate dehydrogenase  (U/L) | β0 | 184.00 | 2.55 | 72.14 | <0.001 | 179.00 | 189.00 |  | 185.34 | 2.60 | 71.33 | <0.001 | 180.24 | 190.43 |
|  | β1 | 0.01 | 0.01 | 0.97 | 0.33 | -0.01 | 0.02 |  | 0.01 | 0.01 | 1.91 | 0.06 | 0.00 | 0.03 |
|  | β2 | -3.88 | 3.67 | -1.06 | 0.29 | -11.08 | 3.32 |  | -6.75 | 3.58 | -1.89 | 0.06 | -13.78 | 0.27 |
|  | β3 | 0.01 | 0.01 | 0.77 | 0.44 | -0.01 | 0.03 |  | 0.00 | 0.01 | -0.01 | 0.99 | -0.02 | 0.02 |
| Alkaline phosphatase  (U/L) | β0 | - | - | - | - | - | - |  | 74.69 | 1.54 | 48.62 | <0.001 | 71.67 | 77.70 |
|  | β1 | - | - | - | - | - | - |  | 0.00 | 0.00 | 1.02 | 0.31 | 0.00 | 0.01 |
|  | β2 | - | - | - | - | - | - |  | -4.31 | 2.33 | -1.85 | 0.07 | -8.89 | 0.26 |
|  | β3 | - | - | - | - | - | - |  | 0.00 | 0.00 | 0.93 | 0.35 | 0.00 | 0.01 |
| FT4  (pmol/l) | β0 | - | - | - | - | - | - |  | 16.30 | 0.40 | 40.56 | <0.001 | 15.51 | 17.09 |
|  | β1 | - | - | - | - | - | - |  | 0.00 | 0.01 | -0.39 | 0.70 | -0.01 | 0.01 |
|  | β2 | - | - | - | - | - | - |  | -1.07 | 0.59 | -1.82 | 0.07 | -2.24 | 0.09 |
|  | β3 | - | - | - | - | - | - |  | 0.01 | 0.01 | 0.80 | 0.43 | -0.01 | 0.02 |
| STSH  (mIU/L) | β0 | 2.40 | 0.45 | 5.32 | <0.001 | 1.52 | 3.29 |  | 2.09 | 0.24 | 8.88 | <0.001 | 1.63 | 2.56 |
|  | β1 | 0.00 | 0.00 | 0.43 | 0.67 | 0.00 | 0.00 |  | 0.00 | 0.00 | -0.50 | 0.62 | -0.01 | 0.01 |
|  | β2 | -0.59 | 0.65 | -0.91 | 0.37 | -1.86 | 0.69 |  | 0.16 | 0.27 | 0.61 | 0.54 | -0.36 | 0.69 |
|  | β3 | 0.00 | 0.00 | 0.22 | 0.82 | 0.00 | 0.01 |  | 0.00 | 0.00 | 0.67 | 0.50 | -0.01 | 0.01 |
| anti tg  (U/ml) | β0 | 46.53 | 7.07 | 6.58 | <0.001 | 32.65 | 60.41 |  | 74.58 | 15.43 | 4.83 | <0.001 | 44.18 | 104.98 |
|  | β1 | -0.03 | 0.03 | -1.03 | 0.30 | -0.09 | 0.03 |  | -0.45 | 0.19 | -2.41 | 0.02 | -0.82 | -0.08 |
|  | β2 | 9.71 | 9.47 | 1.03 | 0.31 | -8.87 | 28.30 |  | 22.62 | 16.79 | 1.35 | 0.18 | -10.46 | 55.71 |
|  | β3 | -0.01 | 0.04 | -0.40 | 0.69 | -0.09 | 0.06 |  | 0.07 | 0.23 | 0.32 | 0.75 | -0.38 | 0.53 |
| 14C-Urea breath tests  (dpm) | β0 | - | - | - | - | - | - |  | 58.01 | 29.71 | 1.95 | 0.05 | -0.82 | 116.84 |
|  | β1 | - | - | - | - | - | - |  | 1.79 | 2.50 | 0.72 | 0.48 | -3.15 | 6.73 |
|  | β2 | - | - | - | - | - | - |  | 259.50 | 135.73 | 1.91 | 0.06 | -9.25 | 528.26 |
|  | β3 | - | - | - | - | - | - |  | -1.39 | 3.25 | -0.43 | 0.67 | -7.84 | 5.05 |
| AFP  (IU/ml) | β0 | - | - | - | - | - | - |  | 2.59 | 0.50 | 5.22 | <0.001 | 1.61 | 3.58 |
|  | β1 | - | - | - | - | - | - |  | 0.02 | 0.01 | 1.73 | 0.09 | 0.00 | 0.04 |
|  | β2 | - | - | - | - | - | - |  | -0.45 | 0.73 | -0.61 | 0.54 | -1.89 | 1.00 |
|  | β3 | - | - | - | - | - | - |  | -0.04 | 0.03 | -1.34 | 0.18 | -0.10 | 0.02 |
| CEA  (ng/ml) | β0 | 1.15 | 0.10 | 11.19 | <0.001 | 0.95 | 1.35 |  | 1.28 | 0.14 | 8.95 | <0.001 | 1.00 | 1.56 |
|  | β1 | 0.00 | 0.00 | -1.11 | 0.27 | 0.00 | 0.00 |  | 0.00 | 0.00 | 0.62 | 0.54 | 0.00 | 0.01 |
|  | β2 | -0.07 | 0.16 | -0.45 | 0.66 | -0.38 | 0.24 |  | -0.13 | 0.25 | -0.53 | 0.60 | -0.63 | 0.37 |
|  | β3 | 0.00 | 0.00 | 1.30 | 0.19 | 0.00 | 0.01 |  | 0.00 | 0.01 | 0.46 | 0.65 | -0.01 | 0.01 |
| CA153  (U/ml) | β0 | - | - | - | - | - | - |  | 8.73 | 1.40 | 6.25 | <0.001 | 5.95 | 11.51 |
|  | β1 | - | - | - | - | - | - |  | -0.08 | 0.05 | -1.73 | 0.09 | -0.17 | 0.01 |
|  | β2 | - | - | - | - | - | - |  | 0.76 | 1.97 | 0.39 | 0.70 | -3.15 | 4.68 |
|  | β3 | - | - | - | - | - | - |  | 0.16 | 0.11 | 1.45 | 0.15 | -0.06 | 0.37 |
| CA199  (U/ml) | β0 | - | - | - | - | - | - |  | 9.99 | 1.71 | 5.83 | <0.001 | 6.59 | 13.38 |
|  | β1 | - | - | - | - | - | - |  | 0.05 | 0.04 | 1.25 | 0.22 | -0.03 | 0.13 |
|  | β2 | - | - | - | - | - | - |  | -3.66 | 3.74 | -0.98 | 0.33 | -11.06 | 3.75 |
|  | β3 | - | - | - | - | - | - |  | -0.05 | 0.18 | -0.26 | 0.80 | -0.40 | 0.31 |
| CA242  (U/ml) | β0 | 1.86 | 0.19 | 9.95 | <0.001 | 1.49 | 2.23 |  | 1.28 | 0.20 | 6.40 | <0.001 | 0.88 | 1.67 |
|  | β1 | 0.01 | 0.00 | 2.06 | 0.04 | 0.00 | 0.02 |  | 0.00 | 0.00 | 0.06 | 0.95 | -0.01 | 0.01 |
|  | β2 | -0.48 | 0.55 | -0.88 | 0.38 | -1.57 | 0.60 |  | -0.29 | 0.35 | -0.85 | 0.40 | -0.98 | 0.39 |
|  | β3 | 0.00 | 0.01 | -0.32 | 0.75 | -0.02 | 0.02 |  | 0.03 | 0.02 | 1.43 | 0.16 | -0.01 | 0.07 |
| Serum iron  (ng/ml) | β0 | 98.93 | 24.07 | 4.11 | <0.001 | 51.44 | 146.41 |  | 149.83 | 34.00 | 4.41 | <0.001 | 82.00 | 217.65 |
|  | β1 | -0.05 | 0.38 | -0.13 | 0.89 | -0.80 | 0.70 |  | -1.89 | 1.35 | -1.41 | 0.16 | -4.58 | 0.79 |
|  | β2 | -2.22 | 33.77 | -0.07 | 0.95 | -68.84 | 64.41 |  | 83.48 | 58.86 | 1.42 | 0.16 | -33.94 | 200.90 |
|  | β3 | 0.42 | 0.65 | 0.65 | 0.52 | -0.86 | 1.69 |  | -0.54 | 2.68 | -0.20 | 0.84 | -5.90 | 4.81 |
| βHCG  (mIU/m) | β0 | 0.34 | 0.01 | 30.73 | <0.001 | 0.32 | 0.37 |  | 0.33 | 0.01 | 30.18 | <0.001 | 0.31 | 0.35 |
|  | β1 | 0.00 | 0.00 | 0.21 | 0.84 | 0.00 | 0.00 |  | 0.00 | 0.00 | 0.38 | 0.71 | 0.00 | 0.00 |
|  | β2 | -0.04 | 0.02 | -1.89 | 0.06 | -0.08 | 0.00 |  | 0.00 | 0.03 | -0.08 | 0.93 | -0.07 | 0.06 |
|  | β3 | 0.00 | 0.00 | 0.66 | 0.51 | 0.00 | 0.00 |  | 0.00 | 0.00 | -0.79 | 0.44 | 0.00 | 0.00 |
| hGH  (ng/ml) | β0 | 0.88 | 0.14 | 6.20 | <0.001 | 0.60 | 1.16 |  | 0.61 | 0.07 | 8.96 | <0.001 | 0.47 | 0.74 |
|  | β1 | 0.00 | 0.00 | -1.41 | 0.16 | -0.01 | 0.00 |  | 0.00 | 0.00 | 0.92 | 0.36 | -0.01 | 0.01 |
|  | β2 | 0.17 | 0.13 | 1.37 | 0.17 | -0.08 | 0.42 |  | -0.10 | 0.24 | -0.40 | 0.69 | -0.58 | 0.38 |
|  | β3 | 0.00 | 0.00 | -0.46 | 0.65 | -0.01 | 0.00 |  | 0.00 | 0.01 | -0.37 | 0.71 | -0.02 | 0.02 |
| 1. PSA   (ng/ml) | β0 | - | - | - | - | - | - |  | 0.28 | 0.04 | 7.78 | <0.001 | 0.21 | 0.35 |
|  | β1 | - | - | - | - | - | - |  | 0.00 | 0.00 | -1.02 | 0.31 | 0.00 | 0.00 |
|  | β2 | - | - | - | - | - | - |  | -0.08 | 0.04 | -1.84 | 0.07 | -0.16 | 0.01 |
|  | β3 | - | - | - | - | - | - |  | 0.00 | 0.00 | 0.93 | 0.36 | 0.00 | 0.01 |

Note: *CI:* confdence interval, $\beta_{0}$ estimates the FI at the beginning of the time series. $\beta_{1}$ estimates the preinfection trend of FI. $\beta_{2}$ estimates the immediate change in the level of FI after infection. $\beta_{3}$ estimates the after infection trend of FI.

*Abbreviations*: *BMI* Body mass index, *FBG* Fasting blood glucose, *HbA1c* Hemoglobin A1c, *LDL-C* Low-density lipoprotein cholesterol, *FT4* Free thyroxine, *sTSH*: sensitive thyroid stimulating hormone, *AFP* alpha fetoprotein, *CEA* carcinoembryonic antigen, *CA153* Carbohydrate antigen 153, *CA199* Carbohydrate antigen 199, *CA242* Carbohydrate antigen 242, *βHCG* β-human chorionic gonadotropin, *Hgh* Human Growth Hormone, *F-PSA* Free-Prostate Specific Antigen.

**Reference**

[1] BULL F C, AL-ANSARI S S, BIDDLE S, et al. World Health Organization 2020 guidelines on physical activity and sedentary behaviour[J]. Br J Sports Med, 2020, 54(24): 1451-1462.

[2] WATSON N F, BADR M S, BELENKY G, et al. Recommended Amount of Sleep for a Healthy Adult: A Joint Consensus Statement of the American Academy of Sleep Medicine and Sleep Research Society[J]. Sleep, 2015, 38(6): 843-844.

[3] SOCIETY C N. Dietary Guidelines for Chinese Residents (2022)[M]. Beijing: People's Health Publishing House, 2022.
